# 2-Deoxy-2-fluoro-d-glucose metabolism in *Arabidopsis thaliana*

**DOI:** 10.3389/fpls.2015.00935

**Published:** 2015-11-03

**Authors:** Amol Fatangare, Christian Paetz, Hanspeter Saluz, Aleš Svatoš

**Affiliations:** ^1^Mass Spectrometry/Proteomics Research Group, Max Planck Institute for Chemical EcologyJena, Germany; ^2^Biosynthesis/NMR Research Group, Max Planck Institute for Chemical EcologyJena, Germany; ^3^Department of Cell and Molecular Biology, Leibniz Institute for Natural Product Research and Infection Biology – Hans Knöll InstituteJena, Germany; ^4^Biology and Pharmacy Faculty, Friedrich-Schiller-UniversityJena, Germany

**Keywords:** 2-deoxy-2-fluoro-d-glucose, FDG, *Arabidopsis thaliana*, plant, metabolism, F-maltose, FDG-6-phosphate, UDP-FDG

## Abstract

2-Deoxy-2-fluoro-d-glucose (FDG) is glucose analog routinely used in clinical and animal radiotracer studies to trace glucose uptake but it has rarely been used in plants. Previous studies analyzed FDG translocation and distribution pattern in plants and proposed that FDG could be used as a tracer for photoassimilates in plants. Elucidating FDG metabolism in plants is a crucial aspect for establishing its application as a radiotracer in plant imaging. Here, we describe the metabolic fate of FDG in the model plant species *Arabidopsis thaliana*. We fed FDG to leaf tissue and analyzed leaf extracts using MS and NMR. On the basis of exact mono-isotopic masses, MS/MS fragmentation, and NMR data, we identified 2-deoxy-2-fluoro-gluconic acid, FDG-6-phosphate, 2-deoxy-2-fluoro-maltose, and uridine-diphosphate-FDG as four major end products of FDG metabolism. Glycolysis and starch degradation seemed to be the important pathways for FDG metabolism. We showed that FDG metabolism in plants is considerably different than animal cells and goes beyond FDG-phosphate as previously presumed.

## Introduction

2-Deoxy-2-[^18^F]fluoro-d-glucose (^18^FDG) is a radioactive glucose surrogate in which the hydroxyl group at C-2 position is substituted by a positron emitting fluorine [^18^F] radio-isotope. [^18^F] has a half-life (t_1/2_) of 109.8 min. Fluorine has small atomic size and the C-F bond length is comparable to that of C-OH bond. Small atomic radius of fluorine imposes minimal structural constraints in the molecule. Therefore, the resulting ^18^FDG species is able to conjugate with target receptors or enzymes without steric hindrances (Phelps, 2004). ^18^FDG uptake and distribution is found to be similar to that of glucose in animal systems. It is commonly used in medical diagnostics and animal studies to trace uptake and metabolism of glucose in metabolically active tissue such as brain tissue or cancer cells (Som et al., 1980; Alavi et al., 1982; Phelps, 2004; Ung et al., 2007).

^18^FDG is a suitable radiotracer for *in vivo* imaging studies spanning over several hours. In addition, the mean dispersion range of emitted positrons is shortest thus allowing resolution in mm range in positron emission tomography (Sánchez-Crespo et al., 2004). The application of ^18^FDG as a radiotracer has been a well-established technique in animal systems but it has rarely been used in plant imaging experiments. Tsuji et al. (2002) first reported ^18^FDG uptake and distribution in tomato plants (Tsuji et al., 2002). Later, Hattori et al. (2008) described ^18^FDG translocation in intact sorghum plants and suggested that it could be used as a tracer for photoassimilate translocation in plants (Hattori et al., 2008). ^18^FDG has also been used to study glycoside biosynthesis in plants as a measure of plant response to defense induction (Ferrieri et al., 2012). Recently, ^18^FDG has been employed as a radiotracer in plants to study amino-sugar-nitrogen (ASN esp. glucosamine) uptake (Li et al., 2014) or solute transport (Partelová et al., 2014). We have previously shown that the radioactivity distribution pattern observed after ^18^FDG feeding is significantly different than another radiotracer like ^68^Gallium-citrate (^68^Ga-citrate) (Fatangare et al., 2014). ^18^FDG radioactivity distribution was also similar to photoassimilates (Fatangare et al., 2014). There is growing evidence that ^18^FDG could also be used as radiotracer in plant imaging studies to probe sugar dynamics. ^18^FDG application in plant imaging necessitates a successful ^18^FDG tracer kinetics model which could be established after unraveling ^18^FDG translocation and its metabolism in plants. Previous literature describes ^18^FDG radioactivity translocation pattern in plants, however, does not illustrate ^18^FDG metabolism in plant cells.

2-Deoxy-2-fluoro-d-glucose (FDG) uptake and metabolism has been extensively studied in animal cells (McSheehy et al., 2000; Kaarstad et al., 2002; Southworth et al., 2003). Being the glucose analog, FDG is transported into the animal cells via the same transporters as glucose (Higashi et al., 1998; Brown et al., 1999; Avril, 2004; Yen et al., 2004). Upon intracellular uptake, FDG is phosphorylated to FDG-6-phosphate (FDG-6-P) by the action of hexokinase or glucokinase (Sols and Crane, 1954; Bessell et al., 1972; Smith, 2001). Further metabolism of FDG-6-P via the glycolytic pathway was found to be inhibited due to fluorine substitution at C-2 position (Lampidis et al., 2006; Kurtoglu et al., 2007b). It was assumed that FDG-6-P underwent no further metabolism and simply accumulated inside the cell (Bessell and Thomas, 1973; Miller and Kiney, 1981; Reivich et al., 1985; Suolinna et al., 1986).

FDG metabolism in plant cells is not characterized till yet but rather presumed to be similar to animal cells (Hattori et al., 2008). However, FDG metabolism in plants might be quite different from the FDG metabolism in animal cells. Plants photosynthesize sugars as photoassimilates. The photoassimilate flux is regulated through numerous sugar transporters toward specialized organelles like plastids and vacuoles or organs like fruits and tubers for storage or utilization. Because of the complexity of biochemical pathways in plants related to sugar metabolism, it is hard to envisage the metabolic fate of FDG in plant cells. Exploring FDG metabolism in plant leaf tissue is one of the critical aspects of ^18^FDG validation as radiotracer for *in vivo* imaging in plants. Unraveling the FDG metabolism is also essential in correct interpretation of ^18^FDG radiotracer imaging studies in plants.

In present work, we analyzed FDG metabolism in *Arabidopsis thaliana* (*A. thaliana*) leaf cells using stable fluorine [^19^F] labeled FDG (^19^FDG). We fed ^19^FDG to *A. thaliana* rosette leaves and later analyzed leaf extracts using liquid chromatography coupled to mass spectrometry (LC-MS) and nuclear magnetic resonance spectroscopy (NMR) to elucidate major end products of ^19^FDG metabolism in plants.

## Materials and methods

### Reagent and chemicals

^19^FDG was purchased from Sigma Aldrich (Sigma-Aldrich Chemie GmbH, Munich, Germany). All chemicals and solvents were of analytical grade.

### Plant material and growth conditions

*Arabidopsis thaliana* Col-0 plants were used for all the experiments. *A. thaliana* seeds were stratified for 3 days at 4°C and grown in soil. Vernalized seeds were placed in 10 cm round pots containing wet soil consisting of 80% Fruhstorfer Nullerde™, 10% vermiculite, and 10% sand, fertilized with Triabon (1 g.L^−1^) and Osmocote Exact Mini (1 g.L^−1^) and treated with *Steinernema feltiae*. Plants were placed in a controlled environment growth chamber at 21°C temperature and 60% humidity under short-day conditions. Light of intensity 190–220 μmol.m^−2^.S^−1^ was provided for 12 h followed by 12 h of darkness.

### ^19^FDG leaf application and extraction

Six to seven week old *Arabidopsis thaliana* plants were used for all experiments. Four mature rosette leaves were selected for ^19^FDG application. Leaves were gently scratched at 4 spots on the abaxial surface of leaf laminae using a micropipette tip. Five microliter of ^19^FDG (20 mg.mL^−1^) solution was immediately applied on each scratched spot. For bulk extraction procedures, to provide larger quantities of ^19^FDG metabolites, rosette leaves were scratched at 6–8 spots. The total volume of ^19^FDG solution (20 mg.mL^−1^) was 30 μl on average for each leaf. Plants were kept under standard growth conditions. ^19^FDG applied leaves were cut from the rosette after 4 h and extracted using a modified methanol/chloroform extraction procedure (Gromova and Roby, 2010). Leaves were cut and ground in liquid nitrogen. Chloroform: methanol:water (1 mL: 2 mL: 1 mL) was added to 0.4 g of ground leaf sample. The mixtures were sonicated in ultrasonic bath (Merck, Eurolab NV, Belgium) for 15 min at room temperature. After sonication, samples were centrifuged at 4000 g for 20 min at 4°C. Supernatants were stored in glass vials at −80°C until further analysis. Samples were analyzed by LC-MS on a LTQ Orbitrap XL™ hybrid ion trap-orbitrap (LTQ-Orbitrap XL) mass spectrometer (Thermo Fisher Scientific GmbH, Bremen, Germany) or by direct infusion-MS on a Q Exactive™ Plus hybrid quadrupole-orbitrap (Q-Exactive Plus) mass spectrometer (Thermo Fisher Scientific GmbH, Bremen, Germany).

### LC-MS and LC-MS/MS measurements

LC-MS data were acquired using a Dionex UltiMate 3000 UHPLC system coupled to a LTQ-Orbitrap XL mass spectrometer. Samples were separated on a Supelco apHera amino column (Supelco Analytical, Bellefonte, Pennsylvania, USA) (15 cm × 4.6 mm, particle size 5 μm) at room temperature. The mobile phase consisted of water (A) and acetonitrile (B). The elution gradient was set as follows: 20% A (0 min), 20% A (0.5 min), 45% A (13 min), 45% A (18 min), 20% A (18.10 min), and 20% A (20 min). The flow rate was 1 mL.min^−1^ and one-quarter of the flow was directed toward MS using a flow splitter. Sample injection volume was 5 μL. Electrospray ionization (ESI) was used in negative ion mode. Capillary temperature was 280°C, and sheath and auxiliary gas flow rates were 40 and 12 arb (arbitrary units), respectively. The sweep gas flow rate was set to 0 arb and source voltage to 4 kV. The capillary voltage and tube lens were set to −41 and −198 V, respectively. During LC-MS measurements, the fourier transform mass spectrometry (FTMS) analyzer resolution was set at 100,000 with full width at half maximum (FWHM) definition and samples were analyzed in full scan mass range of *m/z* 100–800 with the acquisition of profile-type mass spectra.

Samples were further separated on an Acquity UPLC BEH amide column (Waters Corporation, Milford, Massachusetts, USA) (15 cm × 2.1 mm, particle size- 1.7 μm) at room temperature. The mobile phase consisted of water (A) and acetonitrile (B). The elution gradient was set as follows: 20% A (0 min), 20% A (5 min), 50% A (13 min), 50% A (18 min), 20% A (18.10 min), and 20% A (20 min). The flow rate was 0.3 mL.min^−1^ and the injected volume was set at 10 μL. Electrospray ionization (ESI) was used in negative ion mode. Capillary temperature was 275°C, and sheath and auxiliary gas flow rates were 35 and 7 arb (arbitrary units), respectively. The sweep gas flow rate was set at 0 arb and source voltage at 5 kV. The capillary voltage and tube lens were set at -35 and −110 V, respectively. During LC-MS measurements, FTMS resolution was set at 30,000 with FWHM definition and samples were analyzed in full scan mass range of *m/z* 100–800 with the acquisition of profile-type mass spectra.

LC-MS/MS measurements were acquired on a LTQ-Orbitrap XL mass spectrometer. MS/MS measurements of some of the ions were acquired on a Q-Exactive Plus mass spectrometer. Ions were isolated with an isolation window of 1.6 Da. All other parameters were identical to that of LC-MS. MS/MS spectra were acquired at a FT resolution of 15,000 or more at increasing collision energies until fragmentation occurred. Raw data were processed and compared using Thermo Xcalibur version 3.0.63 (Thermo Fisher Scientific GmbH, Bremen, Germany). The mass accuracy error threshold was fixed at 5 ppm.

### LC separation and fractionation of *m/z* 343.1051

One of the compounds of interest (represented by ion *m/z* 343.1051) was further purified for NMR analysis as described here. Samples were firstly separated on apHera amino column (15 cm × 4.6 mm, particle size- 5 μm) at room temperature. The mobile phase consisted of water (A) and acetonitrile (B). The elution gradient was set as follows: 20% A (0 min), 20% A (4 min), 80% A (13 min), 80% A (18.50 min), 20% A (19 min), and 20% A (25 min). The flow rate was 1 mL.min^−1^ and one-quarter of the flow was directed toward MS for the detection of *m/z* 343.1051. ESI-MS parameters were same as described previously for Acquity UPLC BEH amide column. Sample injection volume was 4 μL. Retention time for *m/z* 343.1051 was found to be 5.90 min. We collected LC eluent in the retention time window of 4.75–6.75 min for fractionation of *m/z* 343.1051. The solvent was evaporated from the collected fraction by means of vacuum centrifugation. Dried fraction was resuspended in water and further subjected to LC fractionation using YMC-Pack Polyamine-II column (YMC co., Kyoto, Japan) (25 cm × 4.6 mm, particle size- 5 μm) at room temperature. The mobile phase consisted of water (A) and acetonitrile (B). Elution gradient was set as follows: 20% A (0 min), 20% A (6.50 min), 55% A (18 min), 55% A (24 min), 20% A (24.10 min), and 20% A (30 min). The flow rate was 1 mL.min^−1^ and one-quarter of the flow was directed toward MS for the detection of *m/z* 343.1051. ESI-MS parameters were same as described previously for Acquity UPLC BEH amide column. Sample injection volume was 2 μL. Retention time for *m/z* 343.1051 was found to be 13.50 min. We collected the LC eluate in the retention time window of 13.00–14.00 min for fractionation of *m/z* 343.1051. The solvent was evaporated from the collected fraction by means of vacuum centrifugation. Dried fraction was resuspended in D_2_O and further subjected to NMR analysis for structure elucidation.

### Partial purification of polar ^19^F metabolites for NMR using F-SPE

SiliaPrep Fluorochrom silica gel SPE cartridges (SiliCycle Inc., Quebec City, Quebec, Canada) (3 mL, 500 mg) were equilibrated with distilled water. The concentrated ^19^FDG leaf extract samples were loaded onto the cartridge and eluted from the cartridge with distilled water in sequential fractions of 500 μL. Fractions were vacuum dried, dissolved in 600 μL of D_2_O and analyzed for the presence of ^19^F metabolites using ^19^F-NMR spectroscopy.

### NMR analysis

For NMR structure elucidation, ^1^H- and ^13^C- chemical shift data were acquired on a Bruker Avance AV500 (Bruker BioSpin GmbH, Rheinstetten, Germany) equipped with a 5 mm TCI cryoprobe. Data acquisition was controlled by Bruker Topspin ver.2.1., and pulse programs as implemented were used (^1^H, ^13^C, ^1^H-^1^H dqfCOSY, ^1^H-^13^C HSQC, and ^1^H-^13^C HMBC). Selective TOCSY experiments were accomplished using a pulse sequence as suggested in Thrippleton and Keeler (2003). For probing entire sugar spin systems, the mixing time was set to 200 ms and ^3^J_HH_ correlations were probed by adjusting the mixing time to appropriate values (20–35 ms). For selective irradiation a Gaussian inversion pulse tailored to the respective signal width was used. Selective NOESY experiments were carried out using a Q3 Gaussian pulse cascade and a mixing time of 1.5 s. The samples examined were dissolved in D_2_O and ^1^H-chemical shift data were referenced to the residual solvent peak at 4.70 ppm. ^13^Cchemical shift data were left uncorrected. Carrier frequencies were carefully adjusted to 500.130 MHz for ^1^H-NMR measurements and 125.758 MHz for ^13^C-NMR measurements, respectively.

For ^19^F- and ^31^P-NMR measurements, all 1D and 2D experiments were carried out on a Bruker Avance AV400 spectrometer using a 5 mm BBFO probe. Standard pulse programs as implemented in Bruker TopSpin ver.2.1 were used. All experiments were recorded at 25°C (298 K). Prior to measurements, the carrier frequency was tuned to 376.498 MHz for ^19^F-, 161.976 MHz for ^31^P- and 400.130 MHz for ^1^H-NMR experiments, respectively. ^19^F-NMR spectra were recorded with inverse gated ^1^H-decoupling using a spectral resolution of 256 k data points. The interpulse delay was set to 1 s. One thousand twenty-four scans were applied. Data were processed with a resolution of 128 k and linear back prediction using 32 coefficients, the exponential line broadening was set to 5 Hz. Chemical shifts were referenced to an external standard of neat C_6_F_6_ at −164.9 ppm. ^31^P-NMR spectra were recorded with power-gated ^1^H-decoupling and a spectral resolution of 32 k data points. The interpulse delay was set to 1 s. Data processing was accomplished with 32 k data points and an exponential line broadening of 3 Hz. Chemical shifts were referenced to an external standard of diluted H_3_PO_4_ in D_2_O at 0 ppm. ^1^H-^31^P HMBC-NMR spectra were recorded with 4 k data points in F2 and 128 data points in F1, respectively. Two hundred fifty-six scans were applied. For processing, data were zero-filled to a 2 × 1 k matrix.

## Results

### FDG application affected local leaf tissue

**Figure 1:**
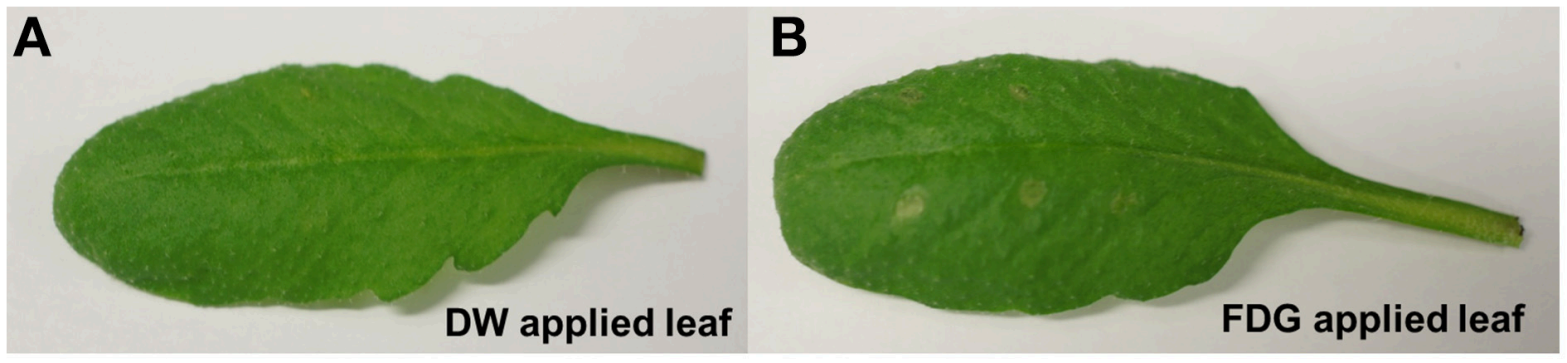
**FDG application affected local leaf tissue**. Mature leaf was scratched on abaxial surface at 6 spots and 5 μL of distilled water (DW) **(A)**, FDG (20 mg.mL^−1^) **(B)** was applied locally on each spot. Leaf pictures were taken after 4 h. Dehydrated spots were visible at FDG application site.

Dehydration was observed at the FDG application site (Figure 1B). It seemed to be appearing from localized water loss arising from osmotic imbalance. On the other hand, distilled water application did not result in dehydration (Figure 1A). Equimolar glucose application also led to the similar dehydration effect (Supplementary Figure 3). The negative effect of FDG on leaf tissue may be a result of high local FDG concentrations and/or FDG cytotoxicity in plant cells.

### Fluorine containing metabolites were characterized by LC-MS and LC-MS/MS analysis

We compared the LC-MS and direct infusion MS spectra manually and looked for characteristic precursor ions which gave rise to fragment ions with HF or ^19^FDG neutral losses upon fragmentation. LC-MS and LC-MS/MS results confirmed the presence of 5 different ^19^F containing metabolites (Table 1). In total, we putatively identified above ^19^F containing metabolites as FDG (*m/z* 181.0513), F-gluconic acid (*m/z* 197.0464), FDG-X-P (*m/z* 261.0180), F-disaccharide (*m/z* 343.1051), and UDP-FDG (*m/z* 567.0434) on the basis of known literature information, their exact mono-isotopic mass (±5 ppm) and MS/MS fragmentation analysis. The three most abundant fluorine containing compounds, FDG, FDG-X-P and F-disaccharide, were collected by means of UPLC/HPLC and a fraction collector. It should be mentioned that in all cases acquired fractions were contaminated with non-fluorinated impurities. For final characterization of the most abundant ^19^F-metabolites FDG-X-P and F-disaccharide, NMR analysis was utilized.

**Table 1:** **List of precursor ions which gave rise to fragment ions with HF or ^19^FDG neutral losses upon fragmentation (underlined MS/MS fragment), their monoisotopic mass, retention time and MS/MS fragmentation**.

**LC column**	**Retention time (min)**	***m/z* (precursor ion)**	**MS/MS fragments**
Supelco apHera amino column	3.54	181.0513	163.0493, 161.0450, 143.0344, 125.0237, 119.0341, 113.0235, 101.0234, 83.0127, 71.0126, 59.0126
Supelco apHera amino column	5.32	343.1051	323.0990, 305.0876, 245.0670, 179.0564
Acquity UPLC BEH amide column	3.80–5.00 (broad and shifting peak)	197.0464	179.0359, 177.0401, 170.0721, 161.0088, 151.0608, 142.9981, 126.9044, 101.0035, 87.0077, 61.9872, 59.0127
Acquity UPLC BEH amide column	4.00–5.60 (broad and shifting peak)	261.0180	243.0073, 241.0116, 223.0010, 204.9903, 199.0009, 145.0278, 138.9797, 96.9686, 78.9579
Acquity UPLC BEH amide column	8.90–10.00 (broad and shifting peak)	567.0434	384.9843, 322.9735, 302.9677, 305.0179, 272.9571, 261.0181, 158.9248, 78.9579

#### *m/z*: 181 (FDG)

Retention time for ^19^FDG standard was found to be 3.54 min on the Supelco apHera amino column. Measured *m/z* 181.0513 value matched with calculated monoisotopic mass of C_6_H_10_O_519_F^−^ (±5 ppm). *m/z* 181.0513 retention time matched with ^19^FDG standard. Upon fragmentation, *m/z* 181.0513 gave rise to secondary ions *m/z* 163.0493 and 161.0450. The first fragment can be rationalized by H_2_O neutral loss (18.0020) and second by HF neutral loss (20.0063). We putatively identified this ^19^F-metabolite as FDG on the basis of its exact mono-isotopic mass (±5 ppm) and MS/MS fragmentation analysis (Supplementary Figure 5A). Identity was further confirmed by NMR experiments.

#### *m/z*: 343 (F-disaccharide)

Retention time for *m/z* 343.1051 was found to be 5.32 min on the Supelco apHera amino column. Measured *m/z* 343.1051 matched with calculated monoisotopic mass of C_12_H_20_O_1019_F^−^ (±5 ppm). Upon fragmentation, *m/z* 343.1051 gave rise to secondary ions *m/z* 323.0990 and 179.0564. The first fragment can be rationalized by HF neutral loss (20.0061), whereas the other fragment ion was identified as deprotonated hexose (C_6_H_11_O${}_{6}^{-}$) which could be rationalized by C_4_H_9_O_419_F neutral loss. We putatively identified this metabolite as ^19^F-disaccharide on the basis of its exact mono-isotopic mass (±5 ppm) and MS/MS fragmentation analysis (Supplementary Figure 5D). This compound was purified and subjected to NMR analysis.

#### *m/z*: 197 (2-deoxy-2-fluoro-gluconic acid)

Retention time for *m/z* 197.0464 was varying in the range of 3.80–5.00 min on the Acquity UPLC BEH amide column. Measured *m/z* 197.0464 matched with calculated monoisotopic mass of C_6_H_10_O_519_ F^−^ (±5 ppm). Upon fragmentation, *m/z* 197.0464 gave rise to secondary ions *m/z* 179.0359 and 177.0401. The first fragment can be rationalized by H_2_O neutral loss (18.0105) and second by HF neutral loss (20.0063). We putatively identified this ^19^F- metabolite as 2-deoxy-2-fluoro-gluconic acid (F-gluconic acid) on the basis of its exact mono-isotopic mass (±5 ppm) and MS/MS fragmentation analysis (Supplementary Figure 5B).

#### *m/z*: 261 (FDG-X-P)

Retention time for *m/z* 261.0180 was varying in the range of 4.00–5.60 min on the Acquity UPLC BEH amide column. Measured *m/z* 261.0180 matched with calculated monoisotopic mass of C_6_H_11_O_8_P^19^F^−^ (±5 ppm). Upon fragmentation, *m/z* 261.0180 gave rise to secondary ions *m/z* 243.0073 and 241.0116. The first fragment can be rationalized by H_2_O neutral loss (18.0107) and second by HF neutral loss (20.0064). We putatively identified this metabolite as FDG-X-P on the basis of its exact mono-isotopic mass (±5 ppm) and MS/MS fragmentation analysis (Supplementary Figure 5C). This compound was purified and subjected to NMR analysis for structure elucidation.

#### *m/z*: 567 (uridine-diphosphate-FDG)

Retention time for *m/z* 567.0434 was varying in the range of 8.90–10.00 min on the Acquity UPLC BEH amide column. Measured *m/z* 567.0434 matched with the calculated monoisotopic mass of C_15_H_22_O_16_N_2_P_219_F^−^ (±5 ppm). Upon fragmentation, *m/z* 567.0434 gave rise to secondary ions *m/z* 384.9843. This fragment can be rationalized by C_6_H_11_O_519_F neutral loss (182.0591). We also found secondary ions of *m/z* 305.0179 and 261.0181 which matched with calculated monoisotopic mass of C_9_H_10_O_8_N_2_P^−^ (±5 ppm) and C_6_H_11_O_8_P^19^F^−^ (±5 ppm) respectively. We putatively identified this ^19^F- metabolite as uridine-diphosphate-FDG (UDP-FDG) on the basis of its exact mono-isotopic mass (±5 ppm) and MS/MS fragmentation analysis (Supplementary Figure 5E).

### NMR analysis revealed FDG-6-P (*m/z* 261.0180), and F-maltose (*m/z* 343.1051) as major end products of ^19^FDG metabolism in *A. thaliana* leaf cells

#### FDG-6-P (2-deoxy-2-fluoro-d-glucose-6-phosphate)

**Figure 2:**
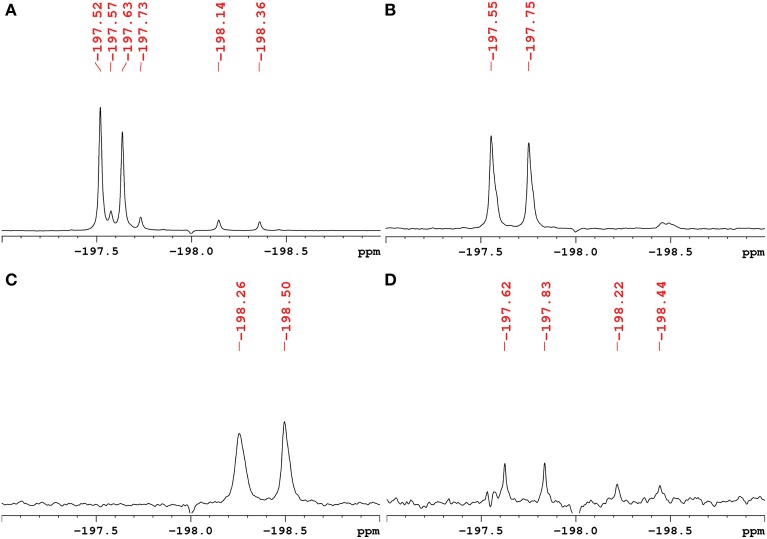
**^1^H-decoupled ^19^F-NMR spectra of fractions containing fluorinated metabolites including chemical shifts**. Signals are referenced to C_6_F_6_ at δ_F_ -164.9. **(A)** Raw extract of *A. thaliana* after FDG administration before separation. The two most intense signals belong to α-FDG (δ_F_ − 197.63) and β-FDG (δ_F_ − 197.52). **(B)** Fraction containing the fluorinated compound α/β-FDG-6-P (*m/z* 261.0180). The α-isomer shows a chemical shift of δ_F_ − 197.75, the β-isomer resonates at δ_F_ − 197.55. **(C)** Fraction containing the fluorinated compound α/β-F-maltose (*m/z* 343.1051). The compound shows signals that appear most deep-field shifted among the identified metabolites (α: δ_F_ − 198.50, β: δ_F_ − 198.26). **(D)** Fraction assumed to contain a fluorinated derivative of gluconic acid (*m/z* 197.0464). The signals indicated likely represent impurities from compounds α/β-FDG-6-P and α/β-F-Maltose.

The exact structure and phosphorylation site of FDG-X-P (*m/z* 261.0180) remained unclear. The semi-purified sample was thus subjected to extensive NMR analysis. ^19^F-NMR spectroscopy revealed FDG-X-P as the most abundant metabolite in the extract, showing two signals at δ_F_ -197.75 (α-FDG-X-P) and δ_F_ − 197.55 (β-FDG-X-P) (Figure 2). The assignment is based on the fact that chemical shift values for α-isomers appear generally shifted toward deeper field compared to the corresponding β-isomers (Southworth et al., 2003). It has to be noted that determined chemical shifts are not in accordance with the literature, which might be either caused by impurities present in the samples and/or due to concentration-dependend shifting. Structure elucidation was therefore based on ^1^H-^1^H and ^1^H-^13^C correlation experiments. Characteristic correlations in the ^1^H-^1^H dqfCOSY spectrum (Supplementary Figure 6) revealed the signals of position 1 at δ_H_ 5.29 (*d*, ^3^J_HH_ = 3.8) (H-1α) and δ_H_ 4.76 (*dd*, ^3^J_HH_ = 7.8/^3^J_HF_ = 2.0) (H-1β), respectively. According to corresponding signals in the ^1^H-^13^C HSQC spectrum, the ^13^C chemical shifts were assigned to δ_C_ 89.6 (*d*, ^2^J_CF_ = 21.2) (C-1α) and δ_C_ 93.5 (*d*, ^2^J_CF_ = 23.6) (C-1β). Since the ^1^H- and ^13^C-NMR spectra were recorded without ^31^P- and ^19^F-decoupling, the extracted coupling patterns revealed the presence of FDG-6-P.

**Figure 3:**
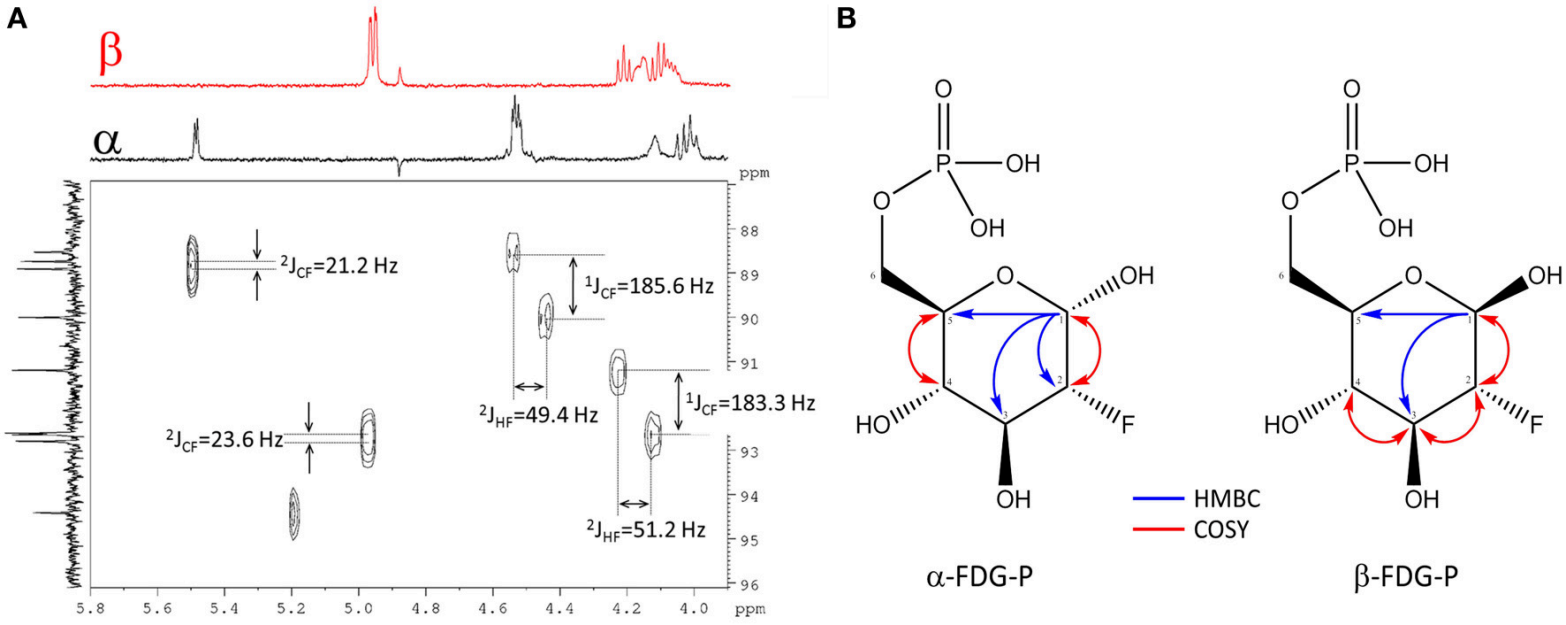
**Assignment of FDG-6-P**. **(A)** Section of the ^1^H-^13^C HSQC spectrum of FDG-6-P. The red F_2_-projection represents the selective TOCSY spectrum of β-FDG-6-P, the black F_2_-trace belongs to α-FDG-6-P. The F1-projection shows the ^13^C-NMR spectrum. Coupling constants were extracted from ^1^H- and ^13^C-NMR spectra, respectively. **(B)** 2D-NMR key correlations used for the assignment of FDG-6-P (α-FDG-6-P and β-FDG-6-P forms, respectively). Blue arrows represent ^1^H-^13^C HMBC correlations from H-1_α/β_. Red arrows indicate ^1^H-^1^H COSY key correlations.

For full structure elucidation, selective TOCSY experiments were performed (Supplementary Figure 7). From irradiation of H-1_α/β_ all remaining partners of the spin systems could be extracted. The resulting spectra served as projections for ^1^H-^1^H dqfCOSY, ^1^H-^13^C HSQC and ^1^H-^13^C HMBC spectra. The signal H-2_α_ of FDG-6-P appeared at δ_H_ 4.28 (*ddd*, ^3^J_HH_ = 3.8/9.5 Hz, ^2^J_HF_ = 49.4 Hz) and H-2_β_ appeared at δ_H_ 3.97 (*ddd*, ^3^J_HH_ = 7.8/9.0 Hz, ^2^J_HF_ = 51.2 Hz), both signals showed an additional splitting due to the coupling to the fluorine substituent through two bonds. The corresponding ^13^C chemical shifts also showed a splitting because of coupling through one bond to the fluorine substituent. The signal C-2_α_ resonated at δ_C_ 90.1 (*d*, ^1^J_CF_ = 185.8 Hz), while C-2_β_ resonates at δ_C_ 92.7 (*d*, ^1^J_*CF*_ = 183.3 Hz) (Figure 3A). From crosspeaks in the ^1^H-^1^H dqfCOSY and ^1^H-^13^C HMBC spectra the chemical shifts of the positions H-3_α/β_ and C-3_α/β_ could be extracted (Figure 3B). H-3_α_ resonates at δ_H_ 3.82 (*ddd*, ^3^J_HH_ = 9.5/9.5 Hz, ^3^J_HF_ = 13.0 Hz) and the corresponding carbon signal C-3_α_ appears at δ_C_ 70.8 (*d*, ^2^J_CF_ = 16.4 Hz). H-3_α_ is overlapped with H-5_α_, but considering the signal geometry in the ^1^H-^13^C HSQC spectrum, the multiplicity could be estimated. H-3_β_ appears as well resolved signal at δ_H_ 3.65 (*ddd*, ^3^J_HH_ = 9.0/9.0 Hz, ^3^J_HF_ = 15.0 Hz) and the corresponding C-3_β_ resonates at δ_C_ 73.6 (*d*, ^2^J_CF_ = 17.6 Hz). H-4_α_ resonates at δ_H_ 3.41 (*dd*, ^3^J_HH_ = 9.5/9.5 Hz) and the attached C-4_α_ appears at δ_C_ 68.5 (*d*, ^3^J_CF_ = 8.0 Hz). H-4_β_ resonates at δ_H_ 3.42 (*ddd*, ^3^J_HH_ = 9.5/9.5 Hz) and the corresponding C-4_β_ appears at δ_C_ 68.6 (*d*, ^3^J_CF_ = 7.8 Hz). The proton signals of position 5_α/β_ show considerable broadening and overlap, therefore only the chemical shift value can be extracted. The chemical shift of C-5_α/β_was extracted from the ^1^H-^13^C HMBC spectrum (Figure 3B). H-5_α_ resonates at δ_H_ 3.80 (*m*) with a corresponding carbon signal C-5_α_ at δ_C_70.2 (*d*, ^3^J_CP_ = 6.2 Hz). The signal for H-5_β_ appears at δ_H_ 3.46 (*m*) with a corresponding carbon signal C-5_β_ at δ_C_74.8 (*d*, ^3^J_CP_ = 6.0 Hz). The chemical shifts of H-6_α/β_ are very similar. The signal of the methylene group at position 6 appears at δ_H_ 3.91 (*bs*) for the α-sugar and at δ_H_ 3.87 (*m*) and δ_H_ 3.97 (*m*), respectively, for the β-sugar. The corresponding carbon resonance for C-6_α/β_ appears at δ_C_ 63.5 as broad singlet signal with a half width of 9 Hz. The splitting of the ^13^C-NMR signal of C-5_α/β_ as well as the broadening for C-6_α/β_ is caused by phosphorylation at position 6_α/β_ which was further corroborated by a ^1^H-^31^P-HMBC experiment (Supplementary Figure 8). The chemical shift of the phosphate residue was determined from a ^31^P-NMR experiment to be δ_*P*_0.83 (*bs*). The structures including determined chemical shifts and coupling constants are summarized in Supplementary Figure 9.

#### F-maltose (4-O-(α-d-glucopyranosyl)-2-deoxy-2-fluoro-d-glucopyranose)

The fluorinated disaccharide (*m/z* 343.1051), characterized by high resolution MS, was identified by NMR to be 4-*O*-(α-d-glucopyranosyl)-2-deoxy-2-fluoro-d-glucopyranose (F-maltose). Chemical shift data extracted are in agreement with published results (Tantanarat et al., 2012).

**Figure 4:**
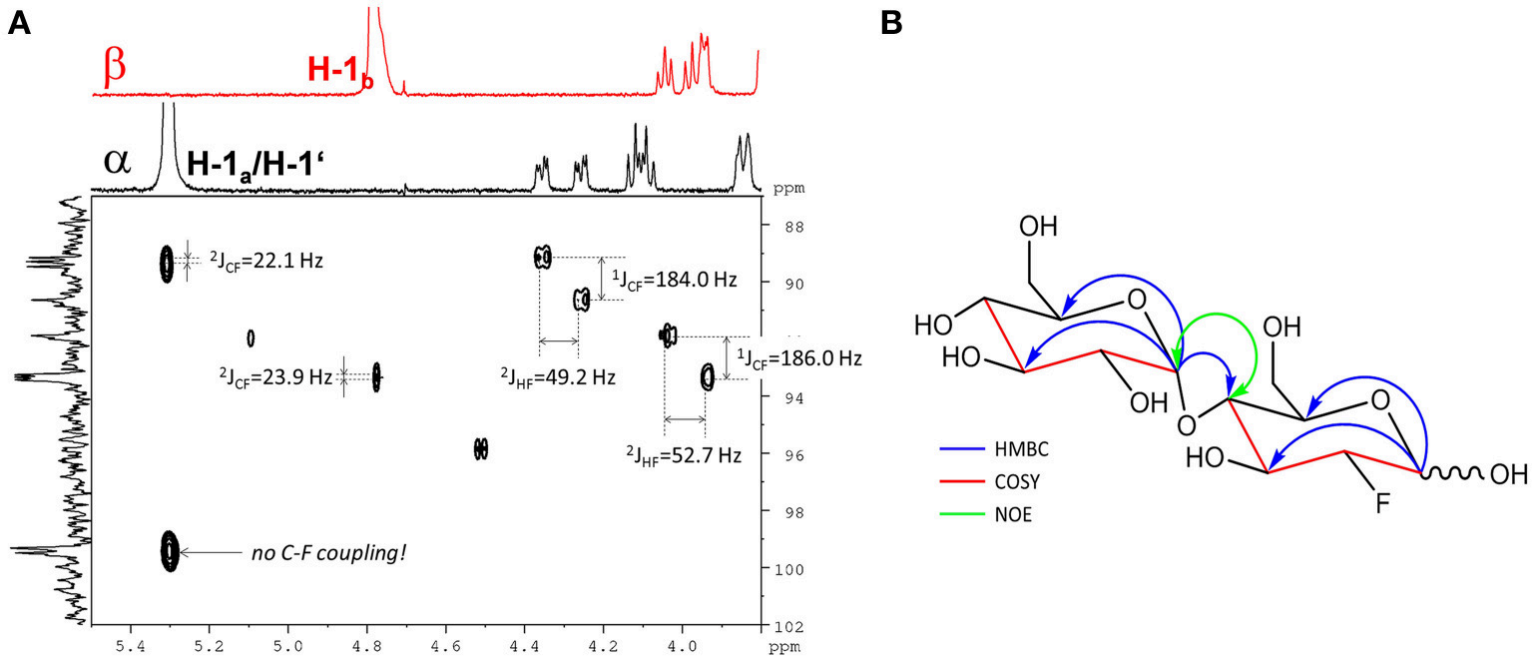
**Assignment of the fluorinated disaccharide. (A)** Detail of the ^1^H-^13^C HSQC spectrum from the fraction containing the fluorinated disaccharide *m/z* 343.1051. Characteristic C-F and H-F couplings are given. Two different shifts (δ_*c*_ 99.3/99.5) for C-1′ appear depending from the configuration of the FDG part. The F2-Projection shows the selective TOCSY spectra for the α/β-FDG part. **(B)** Key correlations used for the structure elucidation of the fluorinated disaccharide *m/z* 343.1051. Blue arrows indicate ^1^H-^13^C HMBC correlations from the position 1 of the respective sugar units. The red parts of the structure indicate for neighboring positions probed by selective COSY experiments. The green double tipped arrow shows the NOE evidence for the α(14) junction between the two sugar units.

The ^19^F-NMR spectrum of the semi-purified sample showed two signals at δ_F_ − 198.26 (β-F-maltose) and δ_F_ − 198.50 (α-F-maltose), revealing this compound to be the second most abundant metabolite formed in *A. thaliana* after FDG administration. The ^1^H-^1^H dqfCOSY spectrum showed characteristic crosspeaks (Supplementary Figure 10). The ^3^J-coupling partners of H-1_α/β_ show the large characteristic split caused by ^2^J_HF_ coupling. Similar to α/β-FDG-6-P, two signals at δ_H_ 5.31 (*d*, ^3^J_HH_ = 3.8 Hz, H-1_α_) and δ_*H*_ 4.78 (*dd*, ^3^J_HH_ = 7.8/^3^J_HF_ = 2.2 Hz, H-1_β_), respectively, represent the anomeric position 1 of the parent FDG structure. A signal overlapping with H-1_α_ was assigned to H-1′. Again, selective TOCSY spectra have been employed to reduce signal overlap from impurities (Supplementary Figure 11). The resulting spectra were used as projections for 2D experiments (^1^H-^1^H dqfCOSY, ^1^H-^13^C HSQC, and ^1^H-^13^C HMBC). Characteristic signals and coupling constants are shown in a section of the ^1^H-^13^C HSQC spectrum (Figure 4A). Structure elucidation was based on information gathered from ^1^H-^13^C-HMBC and a series of selective ^1^H-^1^H COSY and selective ^1^H-^1^H NOESY spectra (Figure 4B). The structures with chemical shifts are summarized in Supplementary Figures 12A,B.

## Discussion

^18^FDG is widely accepted as a radiotracer for glucose in animal studies. However, due to short half-life and radioactivity associated concerns, most of the studies involving FDG metabolism are performed using ^19^FDG. Since both species undergo the same chemical transformations, metabolism results could be extrapolated between the two. We will refer to both species as “FDG” for further discussion of metabolic reactions.

Being a glucose analog, FDG is transported into the animal cells via the same transporters as glucose (Higashi et al., 1998; Brown et al., 1999; Avril, 2004; Yen et al., 2004). It was assumed till recently that upon intracellular uptake, FDG metabolism is restricted to enzymatic phosphorylation, producing FDG-6-P by the action of hexokinases or glucokinases (Bessell and Thomas, 1973; Miller and Kiney, 1981; Reivich et al., 1985; Suolinna et al., 1986). These phosphorylation reactions also introduce the negative charge on the radiotracer preventing its efflux across cell membrane. This leads to increased accumulation of FDG-6-P inside the cell. The assumption that FDG flux is only directed toward FDG-6-P, which leads to intracellular radioisotope accumulation, forms the basis of FDG application as a glucose tracer to study glucose sequestration and utilization. However, numerous studies have demonstrated that FDG metabolism in animal tissue goes beyond FDG-6-P (Kanazawa et al., 1996; McSheehy et al., 2000; Kaarstad et al., 2002; Southworth et al., 2003). These studies list 2-deoxy-2-fluoro-d-mannose (FDM), 2-deoxy-2-fluoro-d-mannose-6-phosphate (FDM-6-P), 2-deoxy-2-fluoro-d-glucose-1-phosphate (FDG-1-P), 2-deoxy-2-fluoro-d-glucose-1,6-bisphosphate, 2-deoxy-2-fluoro-d-mannose-1-phosphate (FDM-1-P), 6-phospho-2-deoxy-2-fluoro-d-gluconolactone, 6-phospho-2-deoxy-2-fluoro-d-gluconate, nucleotide-diphosphate- FDG (NDP-FDG) etc. as metabolic end products of FDG in animal cells (Kanazawa et al., 1996; McSheehy et al., 2000; Bender et al., 2001; Kaarstad et al., 2002; Southworth et al., 2003). It is important to notice that the conversion of FDG to FDG-6-P is the first and foremost step for FDG metabolism in animal tissues and the reported F-metabolites are either situated downstream the FDG-6-P in the glycolytic pathway or arise from it. Thus, net radioactivity accumulated in that tissue still reflects the rate of phosphorylation of FDG and does not challenge the basis of current hypothesis that net FDG uptake by the cell (i.e., total radioactivity acquired by the cell) represents net sugar flux going into glycolysis pathway (Kaarstad et al., 2002).

Among all the studies related to FDG imaging in plants, only few have looked at the metabolism of FDG in plant cells. Thus, FDG metabolism in plants is yet poorly understood. Ferrieri et al. (2012) reported incorporation of FDG in anthocyanin glycoside as a measure of plant defense induction. In this paper, Ferrieri et al. (2012) also suggested the presence of another F-metabolite whose identity was not discovered. Our present work elucidated FDG metabolism in *Arabidopsis thaliana* leaf cells. We have identified the presence of 4 different fluorine-containing metabolites *viz*. F-gluconic acid, FDG-6-P, F-maltose, and UDP-FDG on the basis of known literature information, exact mono-isotopic masses, MS/MS fragmentation and NMR analyses. We also looked for ^19^F-compounds which were previously reported in literature for FDG metabolism in animal tissue. To our surprise, we have not detected 2-deoxy-2-fluoro-6-phospho-d-gluconolactone, 2-deoxy-2-fluoro-6-phospho-d-gluconate or FDG-1,6-bisphosphate (Bender et al., 2001; Kaarstad et al., 2002; Southworth et al., 2003) which were previously reported in various animal studies. We also could not detect the ions for ^19^F-containing major anthocyanin glycosides (*m/z* 1344) in our LC-MS or direct infusion data. This may happen because we acquired and analyzed negative mode MS data whereas anthocyanin glycoside ions may only appear in positive mode MS data.

In a previous paper, we have shown that *A. thaliana* plants take up ^18^FDG from pricked leaf spots and that radioactivity was differentially distributed to various plant parts (Fatangare et al., 2014). *A. thaliana* cell suspension cultures are able to take up FDG from external nutrient and glucose acts as a competitive inhibitor of FDG uptake (Fatangare et al., 2014; Supplementary Figure 1). We also found that FDG uptake by *A. thaliana* cell suspension cultures is severely inhibited by addition of 1 mM HgCl_2_ in the media (Fatangare et al., 2014; Supplementary Figures 1, 2). FDG uptake time course also follows exponential curve (Supplementary Figure 1) similar to glucose uptake rate in glucose starved *Olea europaea* (olive) cells (Oliveira et al., 2002; Conde et al., 2007). This glucose uptake process in olive cells is shown to be mediated by a glucose repressible, H^+^-dependent active saturable transport system which is sensitive to HgCl_2_ (Oliveira et al., 2002; Conde et al., 2007). However, when high external glucose concentrations are present, glucose uptake occurs through a low-affinity, high capacity, protein mediated facilitated transport process which is also sensitive to HgCl_2_ (Conde et al., 2007). On this basis, we hypothesize that FDG is being taken up into the cells via a glucose repressible, H^+^-dependent active saturable transport system at low external FDG concentration and a low-affinity, facilitated-diffusion process at high external FDG concentration.

The H^+^-dependent monosaccharide transporters of olive cells exhibited broad specificity, being able to accept D-glucose, D-fructose, D-galactose, D-xylose, 2-deoxy-d-glucose, and 3-O-methyl-d-glucose (Oliveira et al., 2002). Similarly, numerous monosaccharide transporters (MST) with broad specificity, transporting a range of hexoses and pentoses, have been reported in literature to transport monosaccharides across the plasma membrane (Büttner and Sauer, 2000; Büttner et al., 2000; Büttner, 2007). We think that FDG, being a glucose analog, is transported through one or more of the MST family transporters which are sensitive to HgCl_2_ (Fatangare et al., 2014). Due to broad specificity and highly redundant functional nature of these monosaccharide transporters, it's hard to comment upon which will be the key transporters facilitating FDG uptake in *A. thaliana* leaf cells.

After uptake, FDG is being metabolized to FDG-6-P. This is the first enzymatic conversion in the glycolytic pathway catalyzed by hexokinase. Hexokinase is known to accept FDG as a substrate (Machado de Domenech and Sols, 1980; Muzi et al., 2001). Hexokinase-mediated conversion of FDG into FDG-6-P adds negative charge on influxed FDG and leading to its trapping inside the cell. This maintains the downhill concentration gradient which favors the facilitated transport of FDG into the cell (Printz et al., 1993). This may explain the favorable uptake of external FDG into the cell resulting in high localized accumulation (Fatangare et al., 2014). Upon uptake, FDG was transformed into various metabolites other than FDG-6-P. In future work, we will try to elucidate underlying pathways involved in biosynthesis of these metabolites.

Formation of F-gluconic acid requires spontaneous or enzymatic oxidation of FDG. Buriová et al. (2004) showed formation of F-gluconic acid upon oxidation of FDG (Buriová et al., 2004). We also checked the possibility of a spontaneous oxidation of free FDG into F-gluconic acid during the solvent extraction process. Results showed that there is no formation of F-gluconic acid from the externally added free FDG in the final extracts. This removes the possibility that F-gluconic acid originated as an artifact of spontaneous oxidation process during the sample extraction procedure. In such case, enzymatic dehydrogenation and hydration seems to be plausible way to explain formation of F-gluconic acid. Glucose oxidase (EC 1.1.3.4) or Glucose dehydrogenase (EC 1.1.5.9) could convert glucose to glucono-lactone which upon enzymatic hydration by gluconolactonase (EC 3.1.1.17) can transform into gluconic acid. However, none of the above enzymes have been reported in *Arabidopsis*. Another plausible enzymatic path leading to F-gluconic acid exists but goes through three intermediates like FDG-6-P; 2-deoxy-2-fluoro-d-glucono-1,5-lactone-6-P and 2-deoxy-2-fluoro-d-gluconate-6-P. However, we could not detect these intermediates. At the current moment, we could not comment upon the mechanism of biosynthesis of F-gluconic acid which may be either spontaneous or enzymatic oxidation.

We have observed an F-disaccharide as one of the end products of FDG metabolism in plant leaf tissue. Upon NMR analysis, we identified it to be F-maltose. A cytosolic component of the transitory starch breakdown pathway seems to be most the plausible cause leading to F-maltose biosynthesis *in vivo*. Maltose metabolism in *Arabidopsis* depends upon a disproportionating enzyme and alpha-glucan phosphorylase (Lu et al., 2006). In *Arabidopsis*, cytosolic maltose is mainly metabolized via glucosyltransfer reaction catalyzed by cytosolic glucosyltransferase disproportionating enzyme 2 (DPE2) (EC 2.4.1.25) which transfers one of the glucosyl units of maltose as free glucose and transfers the other to glycogen (Chia et al., 2004; Lu and Sharkey, 2004) or highly branched, soluble heteroglycan (Lu et al., 2006). Reversibly, DPE2 is able to catalyze the transfer of a segment of 1,4-α-d-glucan to a new position in an acceptor, which may be glucose, a 1,4-α-d-glucan (Lin and Preiss, 1988; Kaper et al., 2004; Lu and Sharkey, 2004; Lu et al., 2006; Steichen et al., 2008) or FDG (Tantanarat et al., 2012). FDG could be converted into the F-maltose *in vitro* using DPE2-mediated trans-glycosylation reaction with glycogen acting as a glucosyl donor (Tantanarat et al., 2012). We hypothesize that similar DPE2-mediated trans-glycosylation reaction mechanism must have been involved in biosynthesis of F-maltose. Important to notice here is the conversion of up-taken FDG to F-maltose via DPE2-mediated trans-glycosylation in parallel with FDG conversion to FDG-6-P via hexokinase mediated phosphorylation. Thus, in ^18^FDG experiments, the net radioactivity accumulated in the plant tissue will be reflected by the cumulative rate of FDG conversion by phosphorylation and DPE2-mediated trans-glycosylation. This fact challenges the current hypothesis that net FDG uptake by the plant cell represents net sugar flux going into glycolysis pathway (Kaarstad et al., 2002) and should be taken into consideration while assessing FDG as a tracer for glucose in plant imaging and its uptake in plant cells.

**Figure 5:**
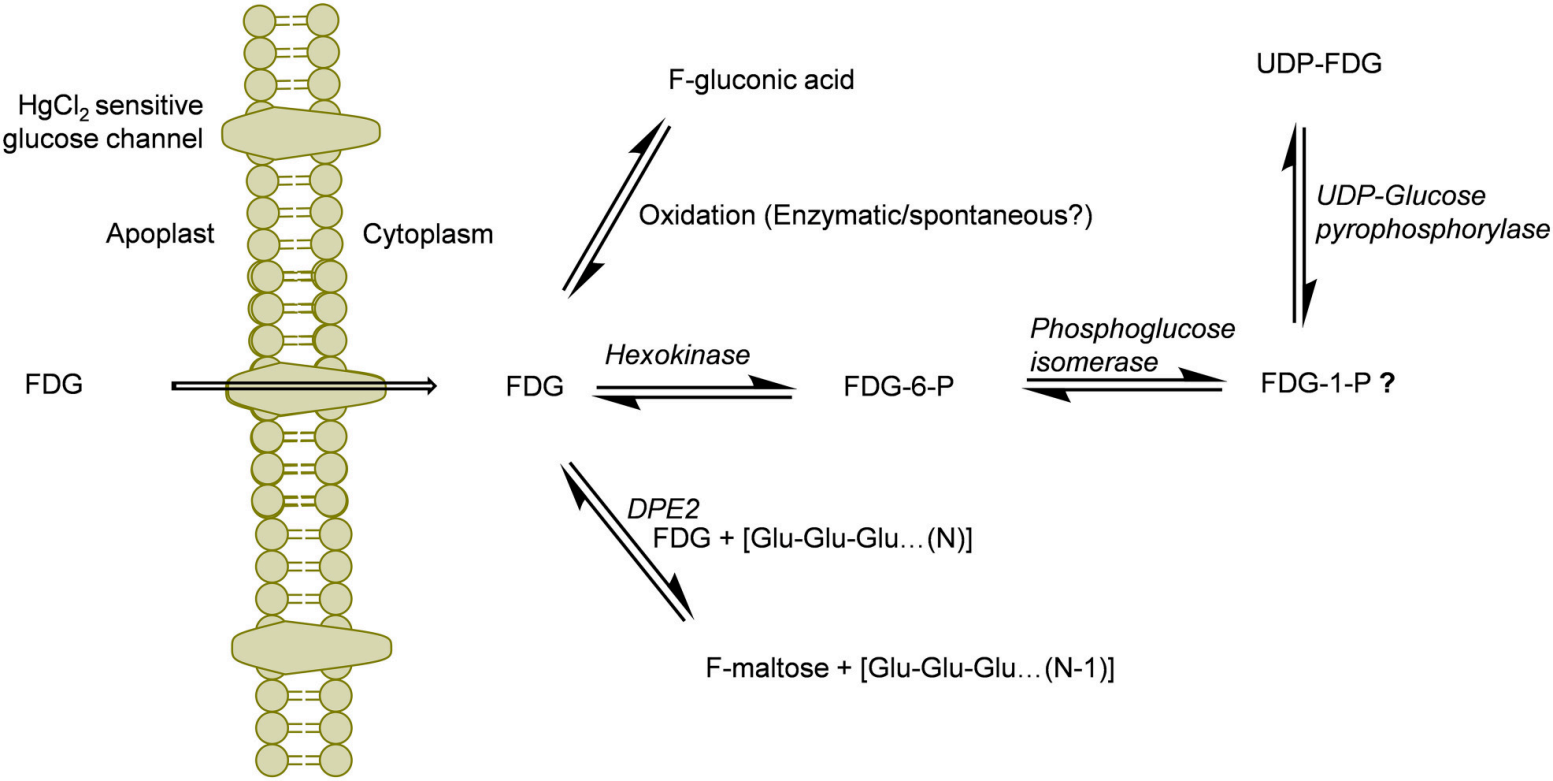
**Schematics of the potential routes of FDG metabolism in plant cell**. FDG, 2-deoxy-2-fluoro-d-glucose; FDG-6-P, FDG-6-phosphate; FDG-1-P, FDG-1-phosphate; UDP, Uridine-diphosphate; F-maltose, 2-deoxy-2-fluoro-maltose; Glu, glucose; *DPE2*, Arabidopsis disproportionating enzyme 2.

Previous studies have demonstrated nucleotide bound forms of FDG (Schmidt et al., 1978; Kanazawa et al., 1996; Southworth et al., 2003). Schmidt et al. (1978) have demonstrated the formation of UDP and GDP derivatives of FDG in yeast and chick embryo cells. NDP-FDG and NDP-FDM were shown to be end products of FDG metabolism in animal tissue (Kanazawa et al., 1996; Southworth et al., 2003). However, assignment of nucleotide species (NDP moiety) was a source of ambiguity in these reports. In our study, we were able to detect only *m/z* 567.0434 which corresponds to UDP-FDG. Thus, we could conclusively point out UDP-FDG as a nucleotide bound form of FDG. Biosynthesis of UDP-FDG has already been described by Kanazawa et al. (1997). We think that similar mechanism exists for the UDP-FDG biosynthesis in plant tissue. The possible UDP-FDG biosynthetic pathway has been shown in Figure 5. This assumption presupposes presence of FDG-1-P as one of the intermediates formed in the process of UDP-FDG biosynthesis. In our studies, we could not detect FDG-1-P as one of the intermediates. We think that FDG-1-P might be present but in very low abundance. UDP-FDG acts as a glucosyl moiety donor in various biosynthetic pathways such as starch, anthocyanin or flavonoid biosynthesis. We think that UDP-FDG may have been involved in biosynthesis of fluorinated anthocyanin (Ferrieri et al., 2012).

Deciphering “Why FDG metabolism is directed toward formation of above mentioned end products” is still unanswered. We think FDG, upon intracellular uptake, will be considered as energy source by the cell and will be fluxed into glycolytic pathway leading to synthesis of FDG-6-P. However, all taken-up FDG could not be metabolized into FDG-6-P as building-up concentration of FDG-6-P inside the cell slow down this bio-transformation through feedback inhibition of hexokinase. FDG-6-P will actually become a catabolic block bringing glycolysis to halt. This has already been shown in hypoxic animal tissue (Datema et al., 1980; Kurtoglu et al., 2007b). This may lead to rest of the free FDG pushed into F-maltose or F-gluconic acid biosynthestic pathways (Figure 5). FDG-6-P may be further transformed into FDG-1-P and finally to UDP-FDG as depicted in Figure 5. Formation of various fluorine-metabolites in plants can be a way of plants to cope up with high intracellular concentration of FDG which is a known glycolytic inhibitor. Thus, biosynthesis of various F-metabolites could also be viewed as utilization of FDG as energy source and a corrective/protective mechanism in the plant cells to counteract its consequences as glycolytic inhibitor.

In our experiment, we visually observed signs of tissue damage at the FDG application site (Figure 1B). FDG, at high local concentrations, may cause cytotoxicity to plant cells, either due to its chemical properties or due to localized osmotic imbalance. It has been shown that FDG interferes with glycolysis, thus resulting in cytotoxicity in hypoxic tumor cells which solely rely on glycolysis for their energy (Maher et al., 2004; Lampidis et al., 2006; Kurtoglu et al., 2007b). In contrast, aerobically growing cells, with functional mitochondria, survive glycolytic inhibition by using carbon sources other than glucose (fats and proteins) to generate ATP via oxidative phosphorylation (Reitzer et al., 1979; McKeehan, 1982; Mazurek et al., 1997; Kurtoglu et al., 2007a). Plant cells might experience similar glycolytic inhibition when fed with FDG. However, for well-aerated leaf tissue, FDG should not cause cytotoxicity. When we applied equimolar glucose solution (20 mg.mL^−1^) as a test, we also observed some extent of localized tissue damage on the leaf (Supplementary Figure 3B). This hints that the observed tissue damage may be caused by localized osmotic imbalance due to external sugar application. FDG also hampers the *N*-linked glycosylation by inhibiting the incorporation of mannose and glucose into lipid-linked oligosaccharides (Datema et al., 1980; Kurtoglu et al., 2007a,b). The inhibition of glycolysis and *N*-linked glycosylation might explain the tissue death/damage we have observed at the site of FDG application. However, this should be confirmed with detailed FDG cytotoxicity studies in plant tissue.

It has been reported that FDG-6-P reversibly epimerizes to FDM-6-P under the action of phosphoglucose isomerase (Kanazawa et al., 1986; Kojima et al., 1988; Pouremad and Wyrwicz, 1991; O'Connell and London, 1995). FDM metabolites such as FDM-1-P, FDM-1,6-bisphosphate and NDP-FDM have been reported in animal tissue (Kanazawa et al., 1996; Southworth et al., 2003). In our study, we mainly focused upon abundant FDG metabolites and could not detect above metabolites. However, presence of corresponding FDM-metabolites and many other F-metabolites reported from animal tissue experiments could not be refuted. We are currently investigating the presence of low abundance F-metabolites in plant tissue and elucidating the ^18^F-radioactivity translocation entity via plant vasculature (Fatangare et al., 2014). In this paper, we reported F-gluconic acid, FDG-6-P, F-maltose and UDP-FDG as four major end products of FDG metabolism in *A. thaliana* leaf tissue and proposed underlying biochemical pathways involved in their biosynthesis (Figure 5). However, these speculations regarding involved biochemical pathways need to be experimentally confirmed. We hope that this work will pave way for discovery of new F-metabolites and will be crucial in understanding and furthering FDG applications in plant imaging.

### Conflict of interest statement

The authors declare that the research was conducted in the absence of any commercial or financial relationships that could be construed as a potential conflict of interest.
