# Supplementary material for: 2-Deoxy-2-fluoro-d-glucose metabolism in Arabidopsis thaliana
Source: Front Plant Sci. 2015 Nov 3;6:935. doi: 10.3389/fpls.2015.00935 (PMC4630959; doi:10.3389/fpls.2015.00935)

**Supplementary data**

**2-Deoxy-2-fluoro-D-glucose metabolism in *Arabidopsis thaliana***

Amol Fatangare^1^, Christian Paetz^2^, Hanspeter Saluz^3,4^, Aleš Svatoš^1^*

^1^ Mass spectrometry/Proteomics research Group, Max Planck Institute for Chemical Ecology, Jena, Germany

^2^ Biosynthesis/NMR research Group, Max Planck Institute for Chemical Ecology, Jena, Germany

^3^ Department of Cell and Molecular Biology, Leibniz Institute for Natural Product Research and Infection Biology – Hans Knöll Institute, Jena, Germany

^4^ Friedrich-Schiller-University Jena, Jena, Germany

* Corresponding author

**Correspondence:**

Dr. Aleš Svatoš,

Director, Mass spectrometry/Proteomics research Group,

Max Planck Institute for Chemical Ecology,

Hans-Knöll-Straße 8, D-07745 Jena, Germany.

Tel: +493641 571700; Fax: +493641571701

Email: [svatos@ice.mpg.de](mailto:svatos@ice.mpg.de)

**Supplementary Figure 1.** ^18^FDG uptake by *Arabidopsis thaliana* cell suspension.

*Arabidopsis thaliana* cell cultures were grown in JPL medium containing sucrose (1.5%). On the day of experiment, cells were suspended in 50 mL JPL medium containing equimolar concentration of mannitol instead of sucrose. Control flasks contained only nutrient media. ^18^FDG solution (1.23 MBq) was added to each flask. Flasks for set-3 and set-4 were added with 100 μM glucose and 1 mM HgCl_2_ respectively prior to ^18^FDG addition. Flasks were kept on a rotary shaker at 60 rpm under normal laboratory light and temperature conditions. 0.5 mL supernatant was collected at sequential time points (30 min, 1 hr, 2 hr, 3 hr, 4 hr, 5 hr time-points) and filtered through a 22 μm filter for removing cell debris. Radioactivity remained in the supernatant was measured measured in counts per second (CPS) using a well counter (Isomed 2100, MED Nuklear-Medizintechnik Dresden GmbH, Dresden, Germany). Two biological replicates (each with three technical replicates) were performed for each set. Radioactivity was decay corrected over time and averaged for two biological replicates. Average decay corrected CPS radioactivity was noted for supernatant.

NOTE: Cell suspension was not completely homogeneous. Small clumps of cells were visible. Thus, cell pellet in 0.5 mL sampling volume could not be extrapolated for total cell pellet activity. Supernatant radioactivity, however, forms a good indicator for FDG concentration remained in the in the flask at time point. Therefore, only supernatant radioactivity but not cell pellet radioactivity was measured at consecutive time points.

Supplementary Table for Fig. 1. ^18^FDG uptake by *Arabidopsis thaliana* cell suspension.

| Flask/time | CTRL (distilled water (DW)+FDG) | Expt (Cell suspension+FDG) | Expt (Cell suspension+100uM Glucose+FDG) | Expt (Cell suspension+1mM HgCl_2_+FDG) |
| --- | --- | --- | --- | --- |
| 0 | 1255.8 | 1279.9 | 1252.3 | 1233.7 |
| 30 | 1259.1 | 973.2 | 1194.2 | 1232.9 |
| 60 | 1239.4 | 787.7 | 1170.4 | 1218.6 |
| 120 | 1268.8 | 555.3 | 1128.7 | 1218.9 |
| 180 | 1269.9 | 390.9 | 1076.6 | 1195.9 |
| 240 | 1280.5 | 285.6 | 1016.2 | 1236.2 |
| 300 | 1264.0 | 221.2 | 955.9 | 1227.2 |

Supplementary Figure 1:


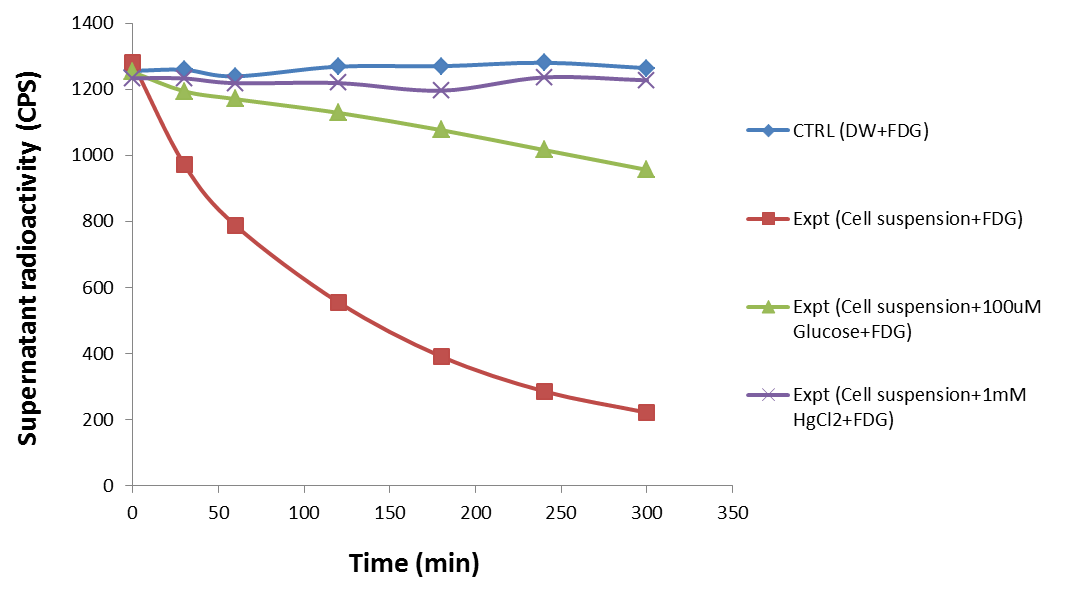


**Supplementary Figure 2.** [Taken from Fatangare et al, 2014]

*Arabidopsis thaliana* cell culture was grown in JPL medium containing sucrose (1.5%). On the day of experiment, cells were suspended in 50 mL JPL medium containing equimolar concentration of mannitol instead of sucrose. Control flasks contained only nutrient media. ^18^FDG solution (1.23 MBq) was added to each flask. Flasks for set-3 and set-4 were added with 100 μM glucose and 1 mM HgCl_2_ respectively prior to ^18^FDG addition. Flasks were kept on rotary shaker at 60 rpm under normal laboratory light and temperature conditions. After 5 hours, the suspensions were filtered through 22 micron filter for cell pellet. Radioactivity accumulated in cell pellet was measured in counts per second (CPS) using a well counter (Isomed 2100, MED Nuklear-Medizintechnik Dresden GmbH, Dresden, Germany). Two biological replicates (each with three technical replicates) were performed for each set. Radioactivity was decay corrected over time and averaged for two biological replicates. Average decay corrected CPS radioactivity was noted for cell pellet.

Supplementary Table for Fig. 2. ^18^FDG uptake by *Arabidopsis thaliana* cell suspension.

| Experimental Set | Average cell pellet radioactivity (CPS) |
| --- | --- |
| Set-1 control (only media) | 22.6 |
| Set-2 Expt (media+suspension+FDG) | 23868.5 |
| Set-3 Expt+glucose(media+suspension+glucose 100μM+FDG) | 8215.5 |
| Set-4 Expt+HgCl_2_ (media+suspension+HgCl_2_ 1mM+FDG) | 604.6 |

Supplementary Figure 2:


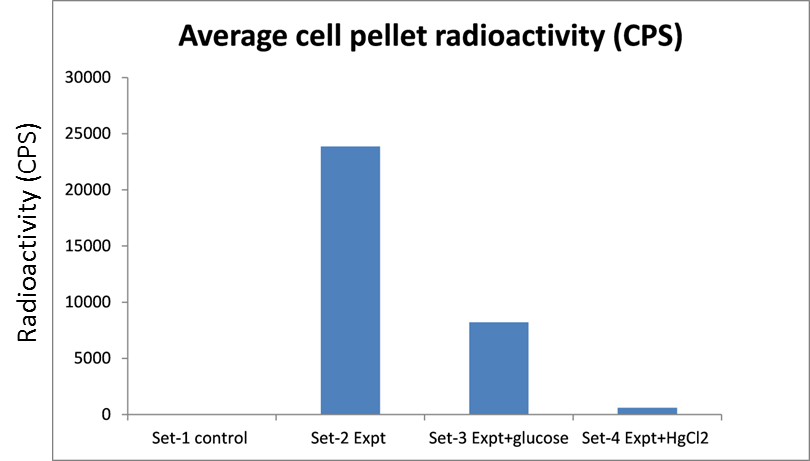


Supplementary Figure 3:

Dehydrated spots were observed at the FDG and glucose application site. Mature leaf was scratched on abaxial surface at 6 spots and 5 μL of FDG (20 mg.mL^-1^) (A), D-glucose (20 mg.mL^-1^) (B) was applied locally on each spot. Leaf pictures were taken after 4 hours. Dehydrated spots were clearly visible at both D-glucose and FDG application sites.


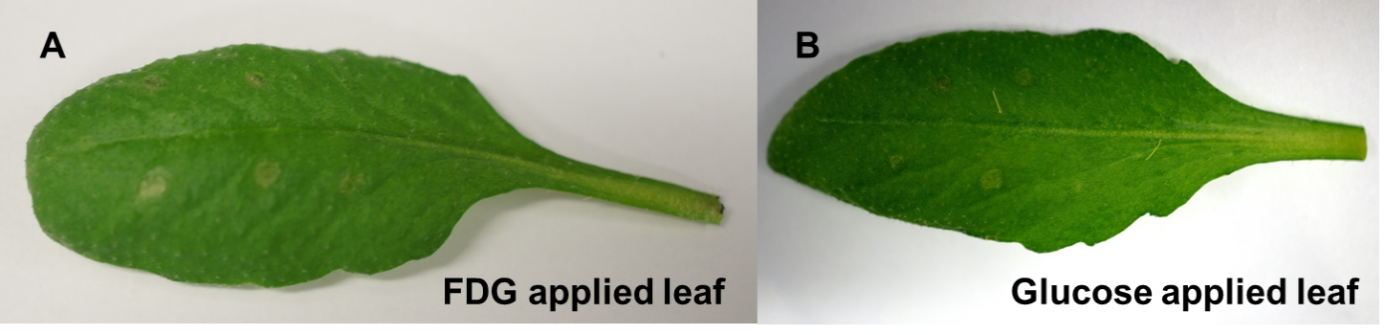


**Supplementary figure 4.**

**TIC and EIC for F-metabolites**  (depicted in Absolute Ion intensity vs RT in minutes)

(A) Total ion chromatogram (TIC) on Supelco apHera amino column

(B) Extracted ion chromatogram (EIC) for *m/z*: 181 (RT: 3.5) on Supelco apHera amino column

(C) Extracted ion chromatogram (EIC) for *m/z*: 343 (RT: 5.3) on Supelco apHera amino column

(D) Total ion chromatogram (TIC) on Acquity UPLC BEH amide column:

(F) Extracted ion chromatogram (EIC) for *m/z*: 197 (RT: 3.8-5.0 broad peak) on Acquity UPLC BEH amide column

(G) Extracted ion chromatogram (EIC) for *m/z*: 261 (RT: 4.0-5.6 broad peak) on Acquity UPLC BEH amide column

(H) Extracted ion chromatogram (EIC) for *m/z*: 567 (RT: 8.9-10.0 broad peak) on Acquity UPLC BEH amide column

**Supplementary figure 5.**

**MS/MS spectra for F-metabolite ions** (depicted in relative ion intensity vs *m/z*)

(A) *m/z*: 181 (FDG)


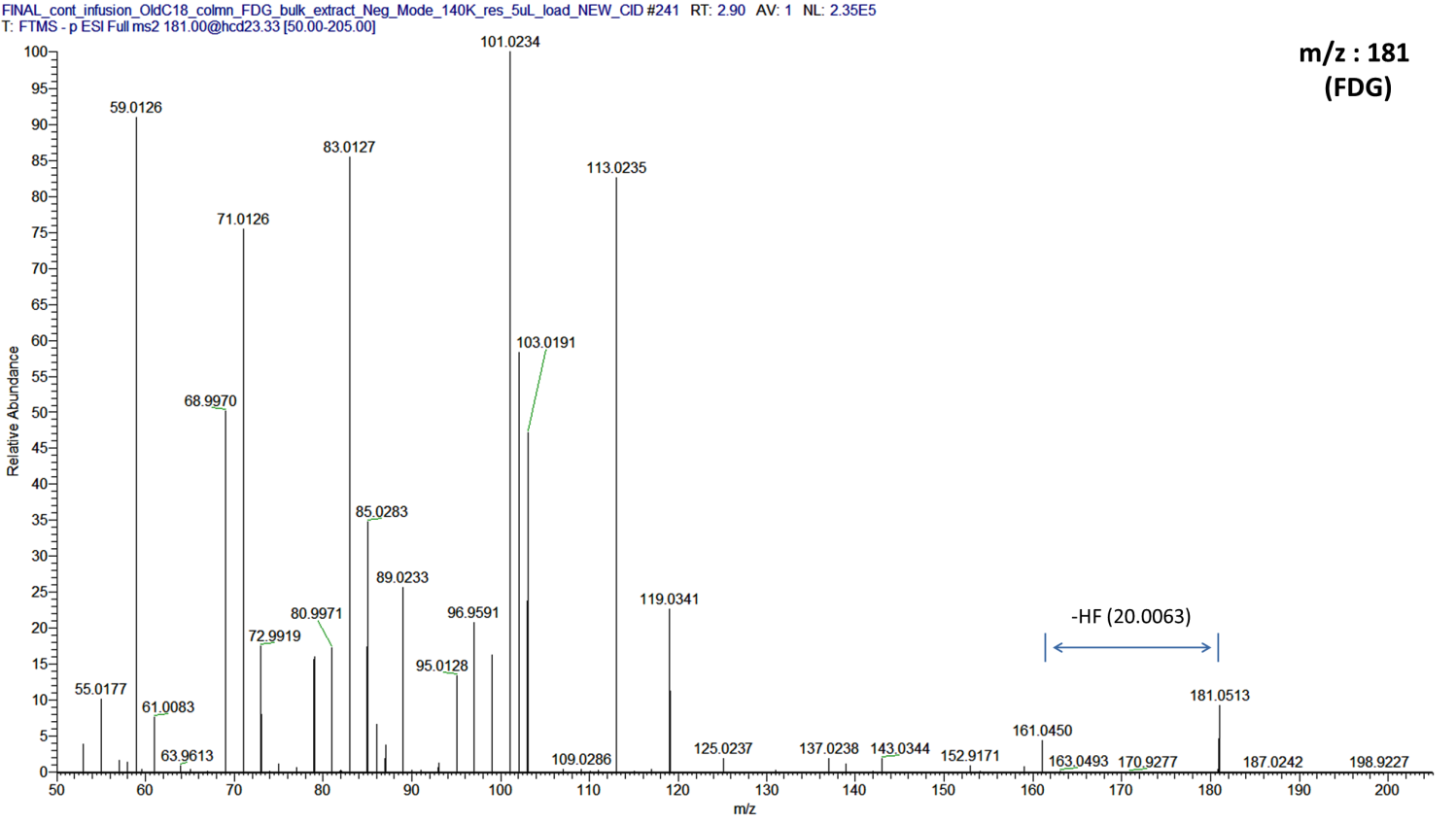


(B) *m/z*: 197 (F-gluconic acid)


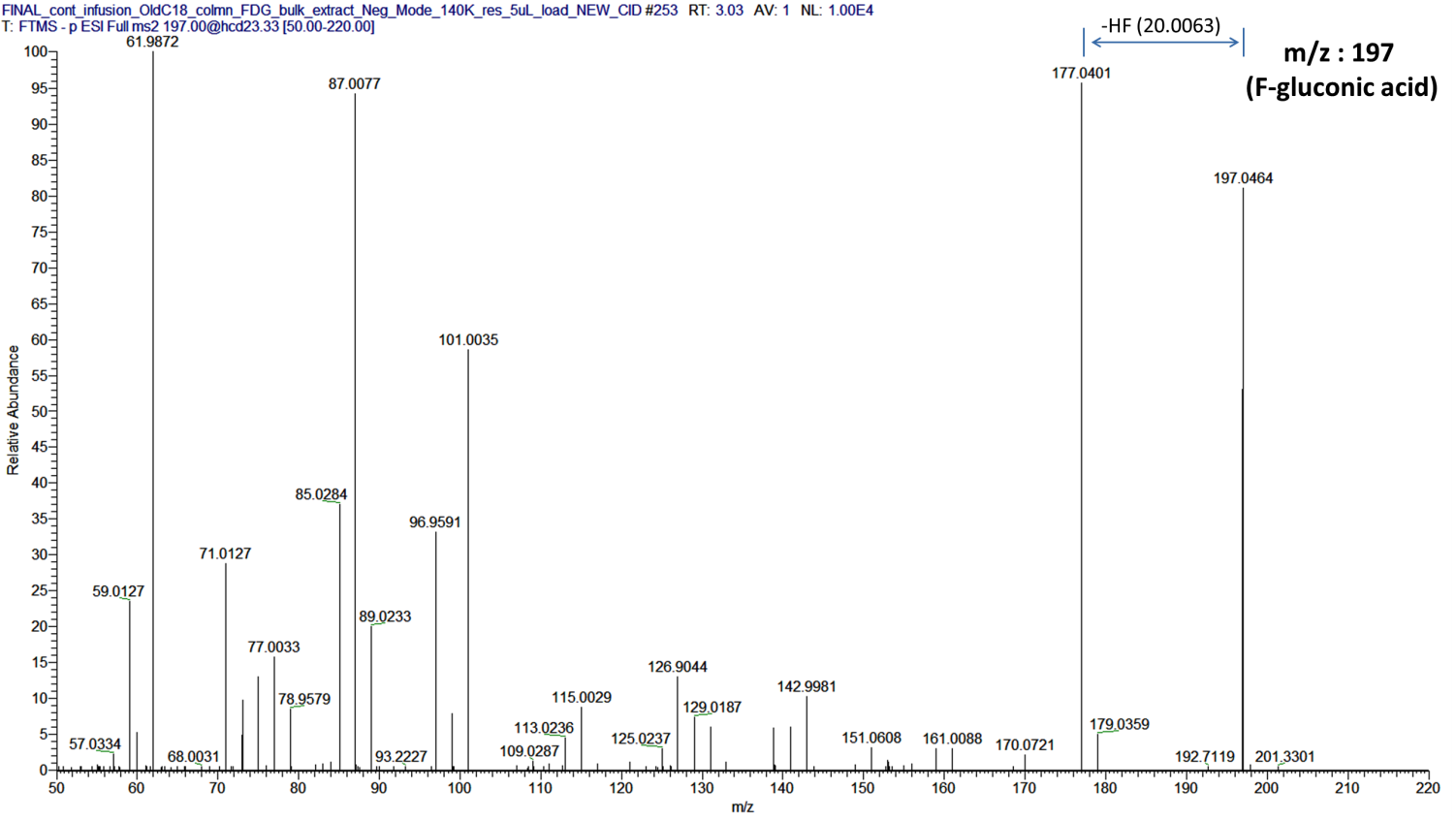


(C) *m/z*: 261 (FDG-6-P)


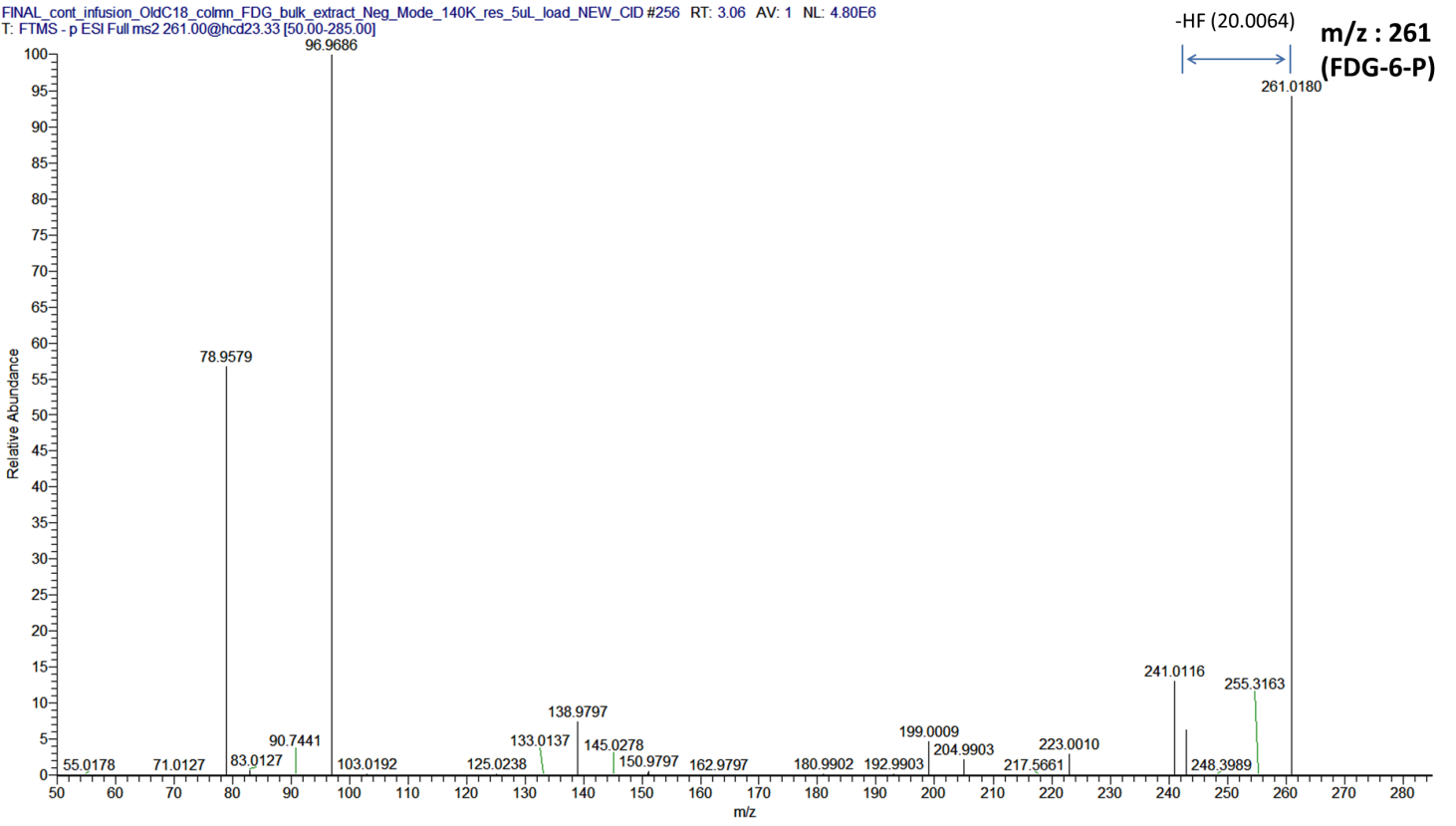


(D) *m/z*: 343 (F-maltose)


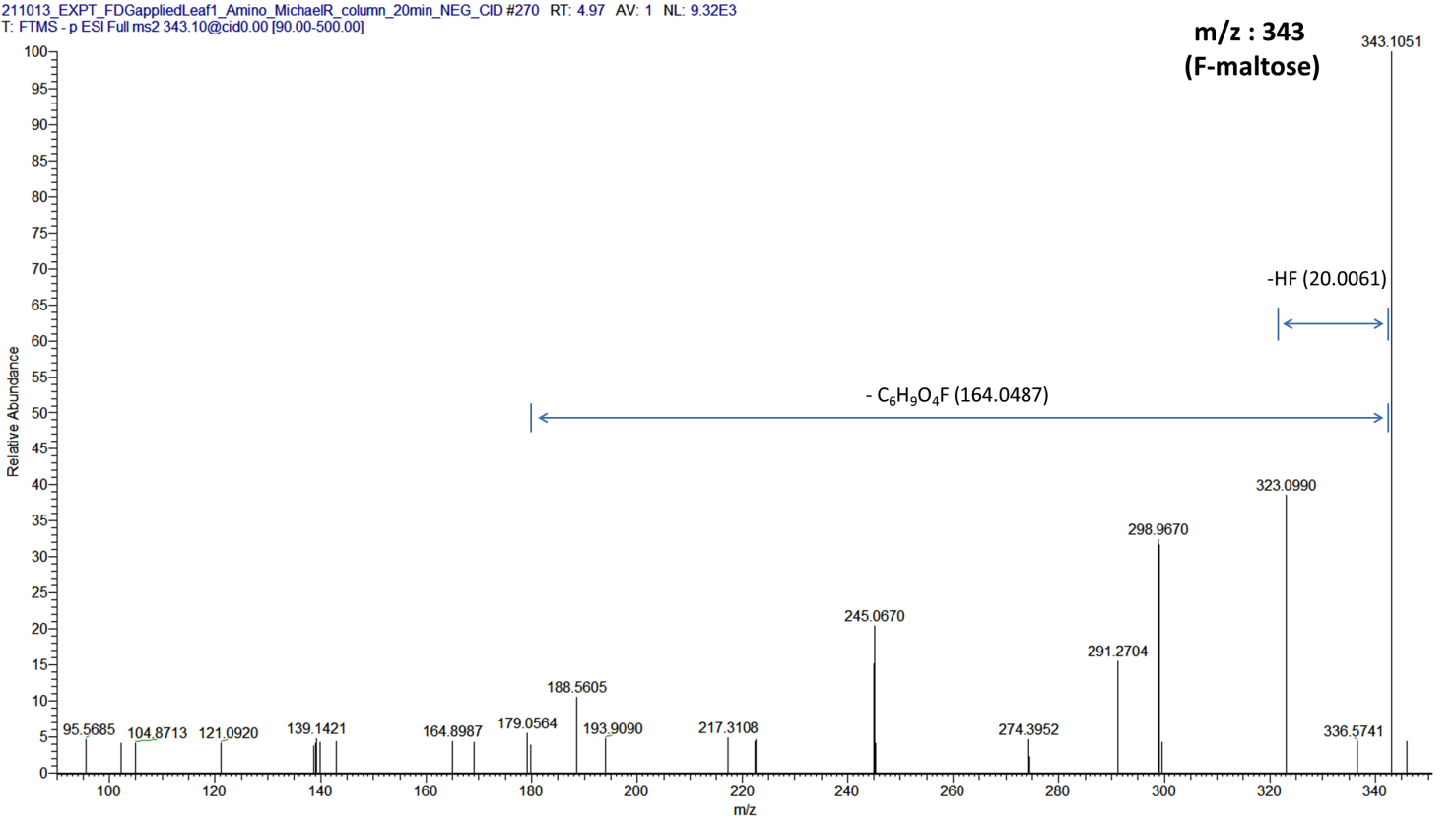


(E) *m/z*: 379 (F-maltose + Cl adduct)


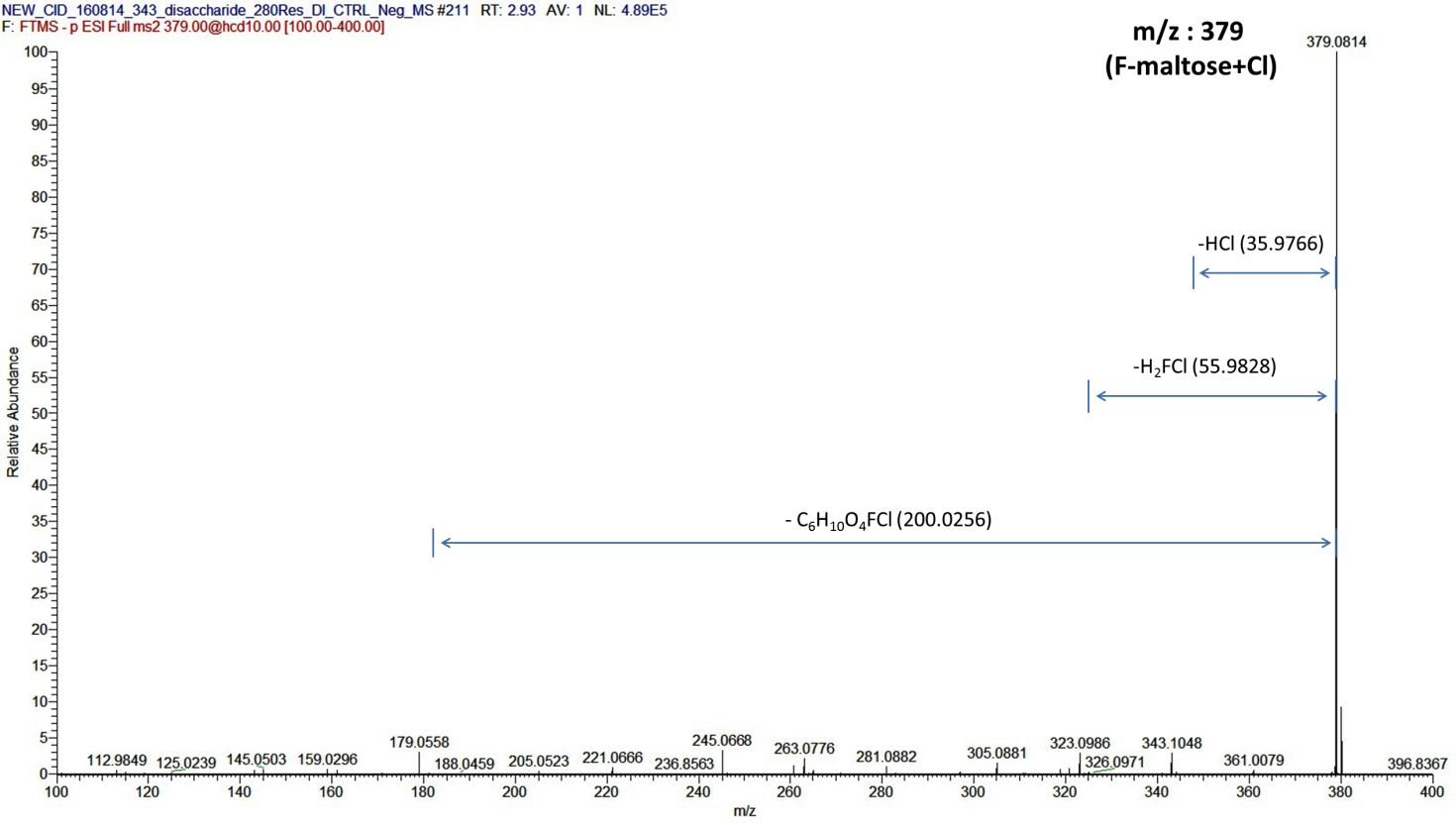


(F) *m/z*: 567 (UDP-FDG)


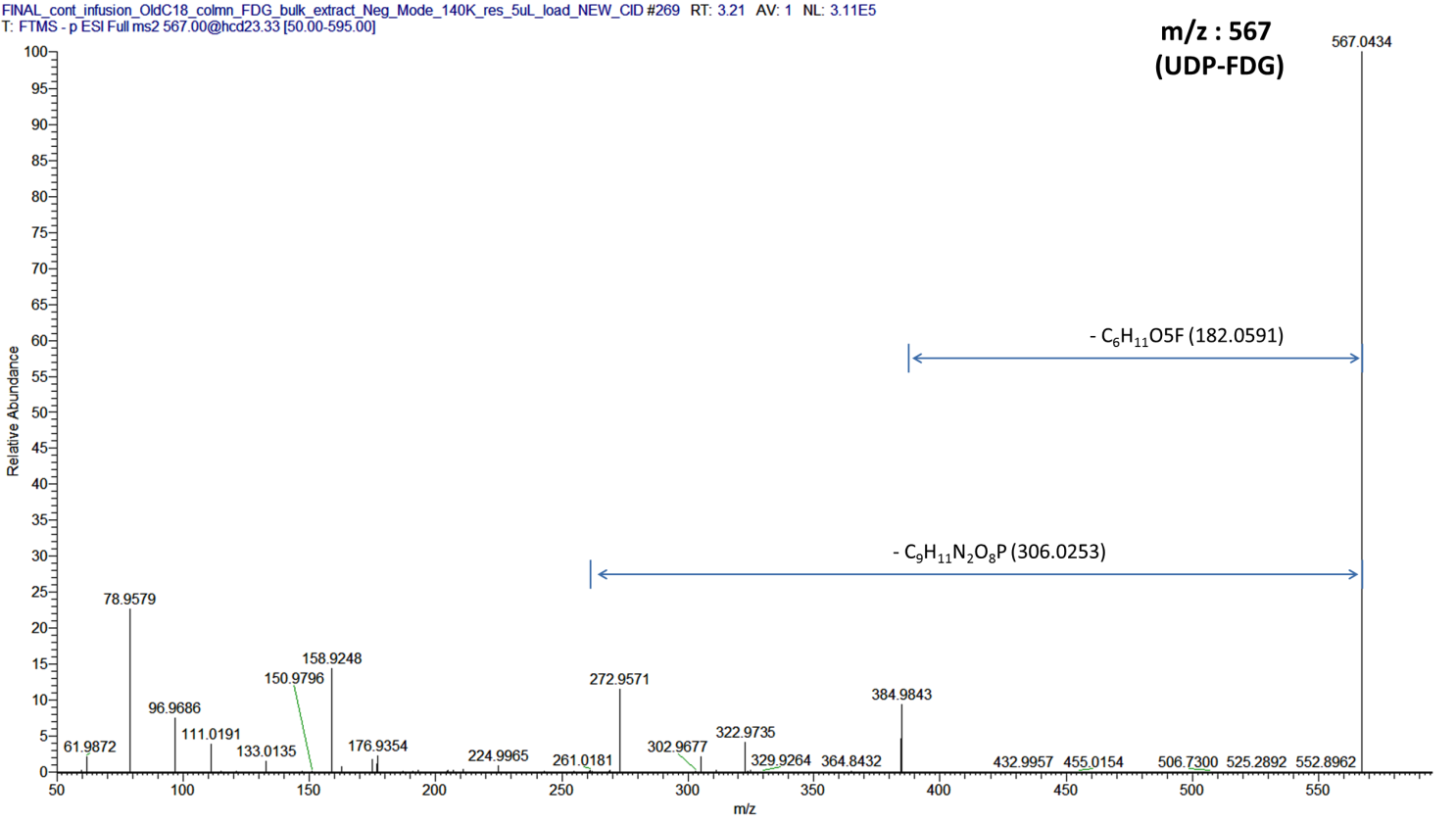


**Supplementary Figure 6**: ^1^H-^1^H dqfCOSY spectrum of the semi-purified fraction of the fluorinated compound *m/z* 261.0180 (FDG-6-P). Key correlations of H-1_α/β_🡪H-2_α/β_ are indicated. The projections show the water-suppressed ^1^H-NMR spectrum.


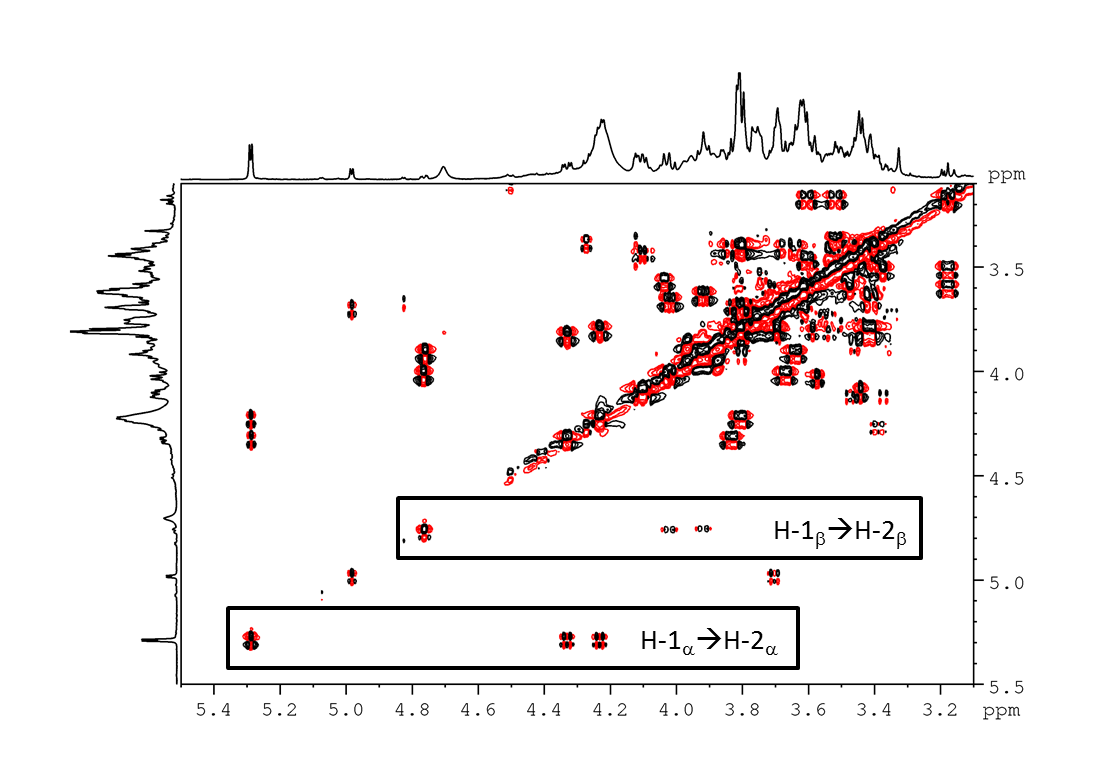


**Supplementary Figure 7**: Comparison of the presaturated ^1^H-NMR spectrum (PURGE, black curve) and the selective TOCSY spectra (SELTOCSY-α/β, red/blue curve) of FDG-6-P.


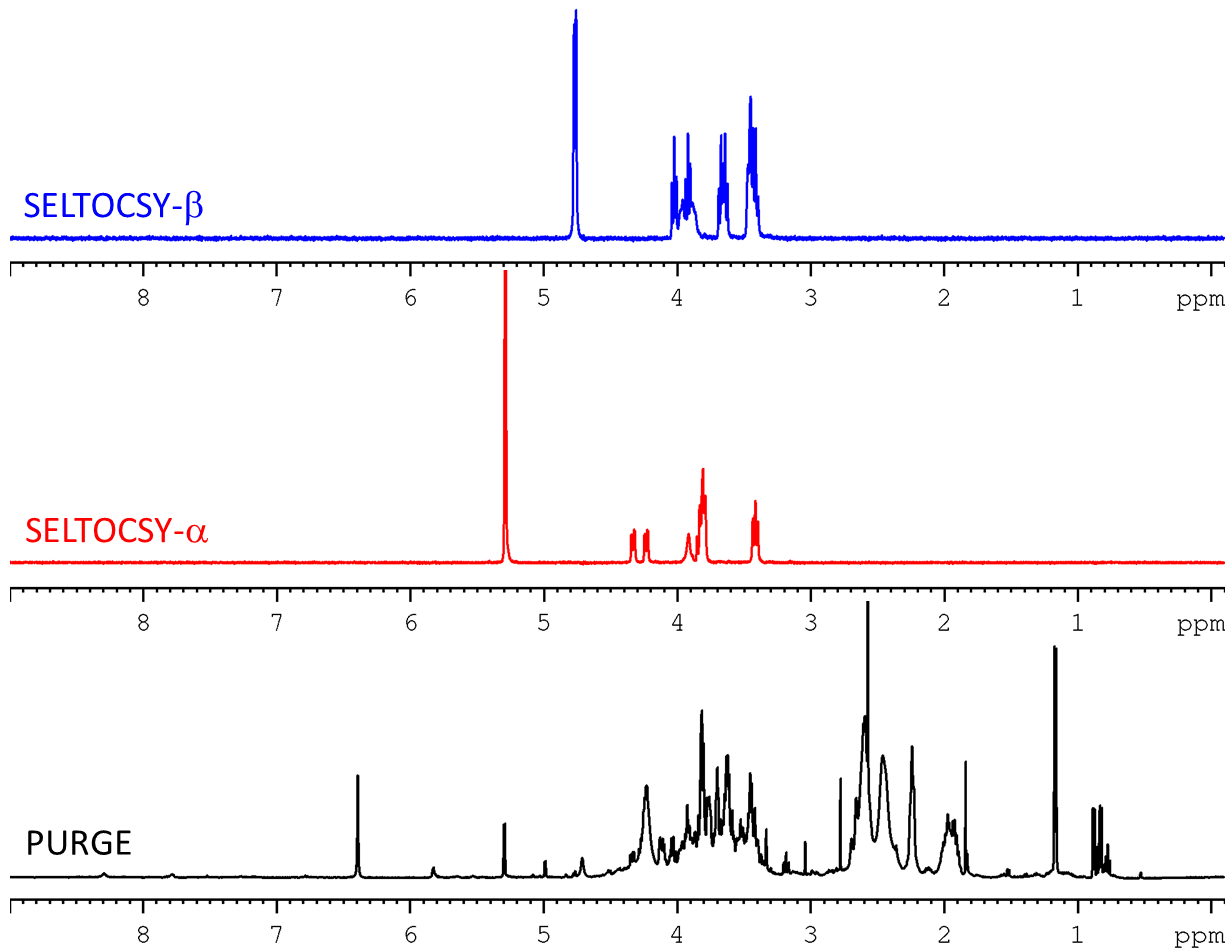


**Supplementary Figure 8**: ^1^H-^31^P-HMBC spectrum of FDG-6-P. A presaturated ^1^H-spectrum serves as F2-projection. The projection in F1 is a ^31^P-NMR spectrum.


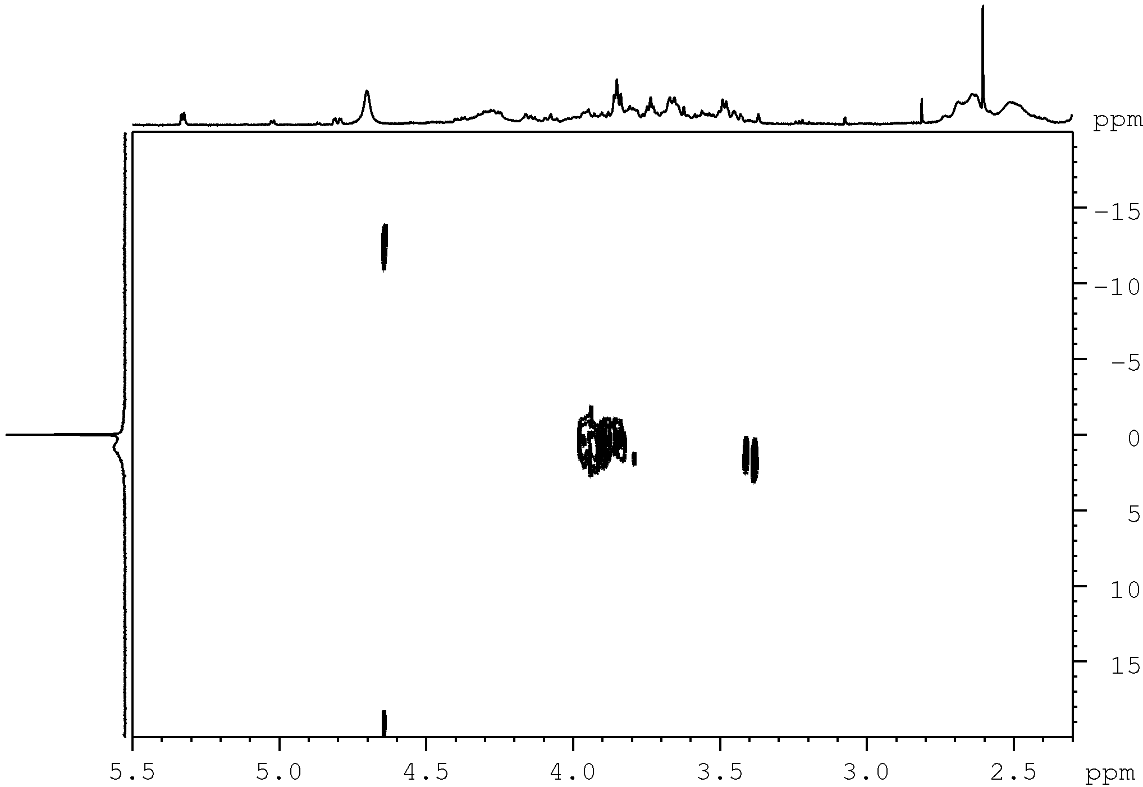


**Supplementary Figure 9**: Structures of α/β-FDG-6-P including chemical shifts, signal multiplicities and coupling constants (^1^H chemical shifts in red, ^13^C chemical shifts in blue, ^19^F chemical shifts in green and ^31^P chemical shifts in magenta. Coupling constants are given in Hz).


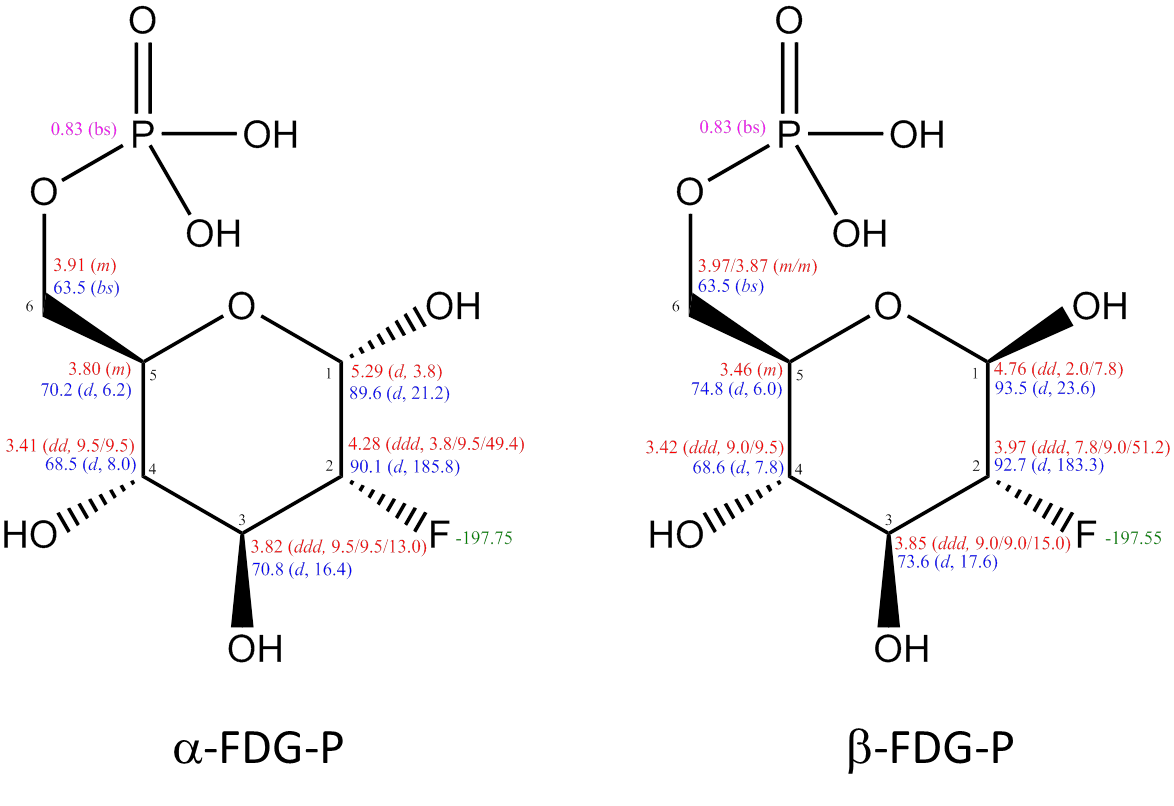


**Supplementary Figure 10**: Presaturated ^1^H-NMR spectrum (using PURGE water suppression) of the fraction containing the fluorinated disaccharide *m/z* 343.1051 (black trace). The red trace shows a selective ^1^H-^1^H TOCSY spectrum derived from irradiating overlapping signals (H-1_α_/H-1’) around δ_H_ 5.30. The blue trace shows a selective ^1^H-^1^H TOCSY spectrum acquired after irradiating H-1_β_ at δ_H_ 4.78.


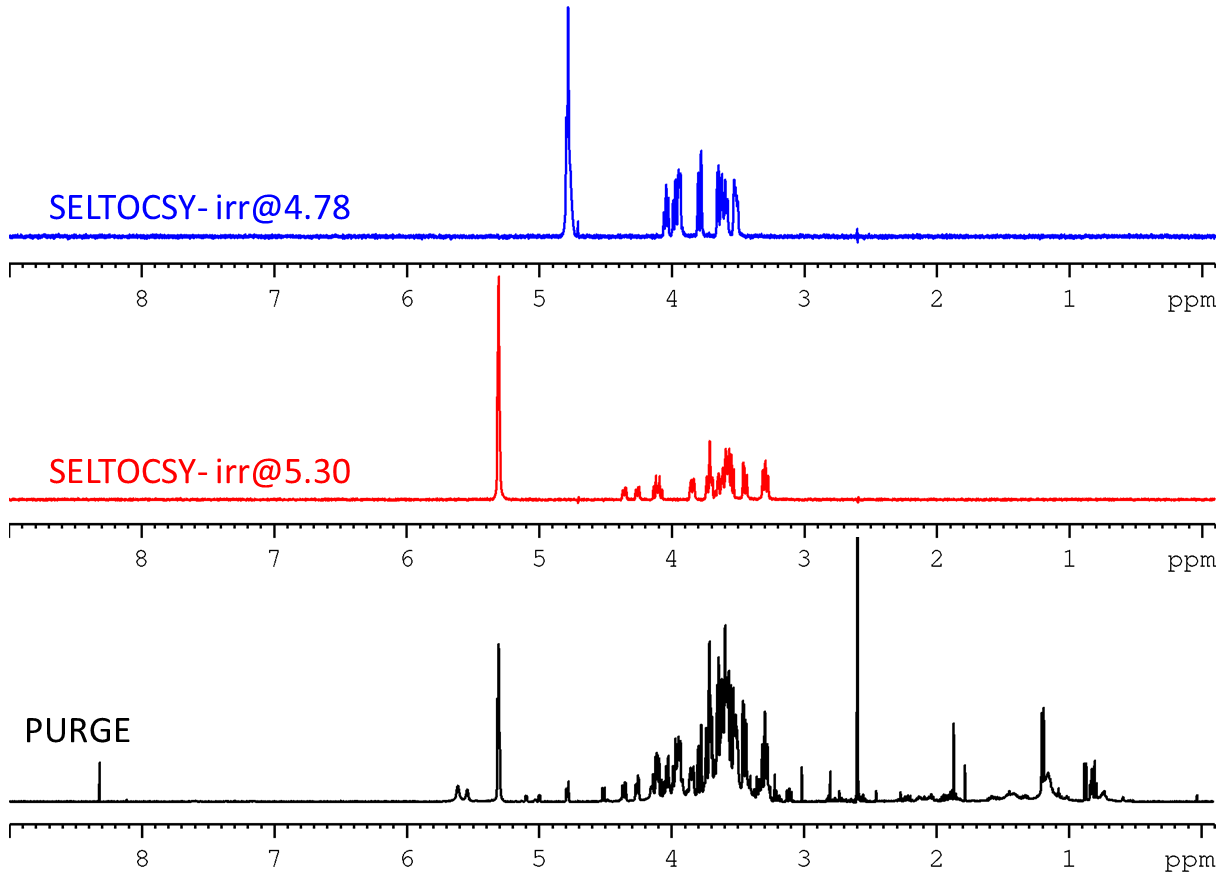


**Supplementary Figure 11**: ^1^H-^1^H dqfCOSY spectrum of the semi-purified fraction of the fluorinated disaccharide (*m/z* 343.1051). Key correlations of H-1_α/β_🡪H-2_α/β_ are indicated. Note the irregular shape of the signal for H-2_β_. The projections show the water-suppressed ^1^H-NMR spectrum.


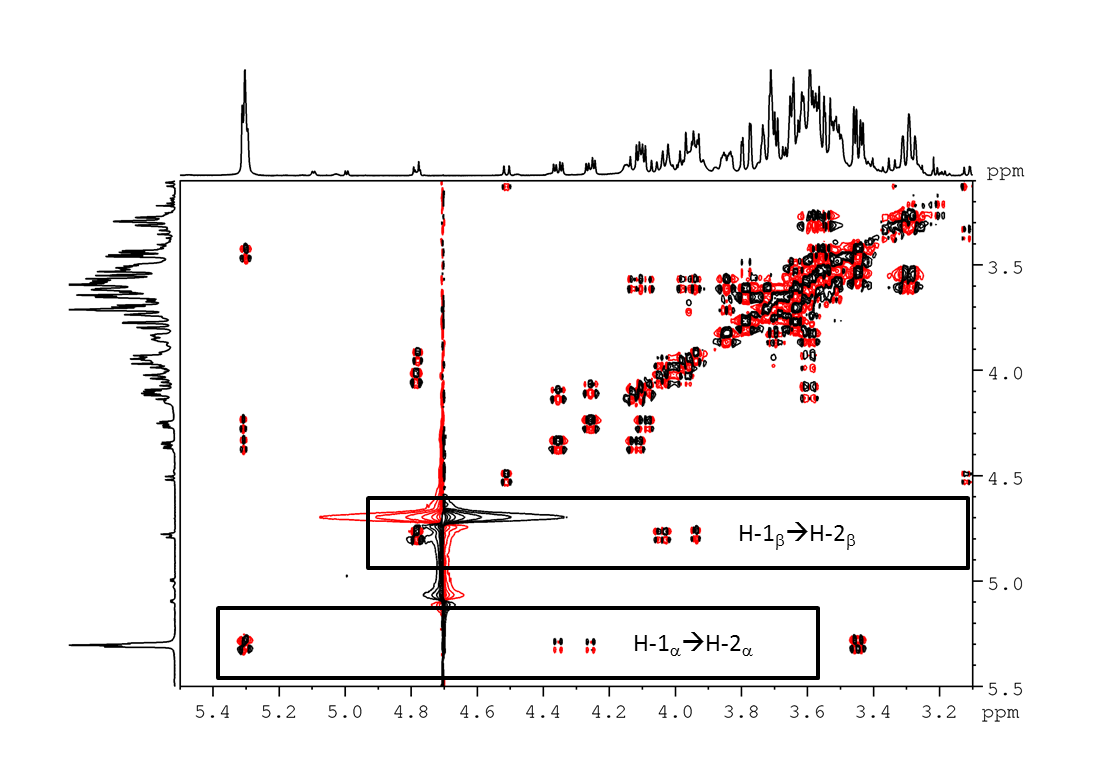


**Supplementary Figure 12**: F-maltose. (A) Structure of α-F-maltose including chemical shifts, signal multiplicities and coupling constants (^1^H chemical shifts in red, ^13^C chemical shifts in blue, ^19^F chemical shifts in green. Coupling constants are given in Hz). (B) Structure of β-F-maltose including chemical shifts, signal multiplicities and coupling constants (^1^H chemical shifts in red, ^13^C chemical shifts in blue, ^19^F chemical shifts in green. Coupling constants are given in Hz).


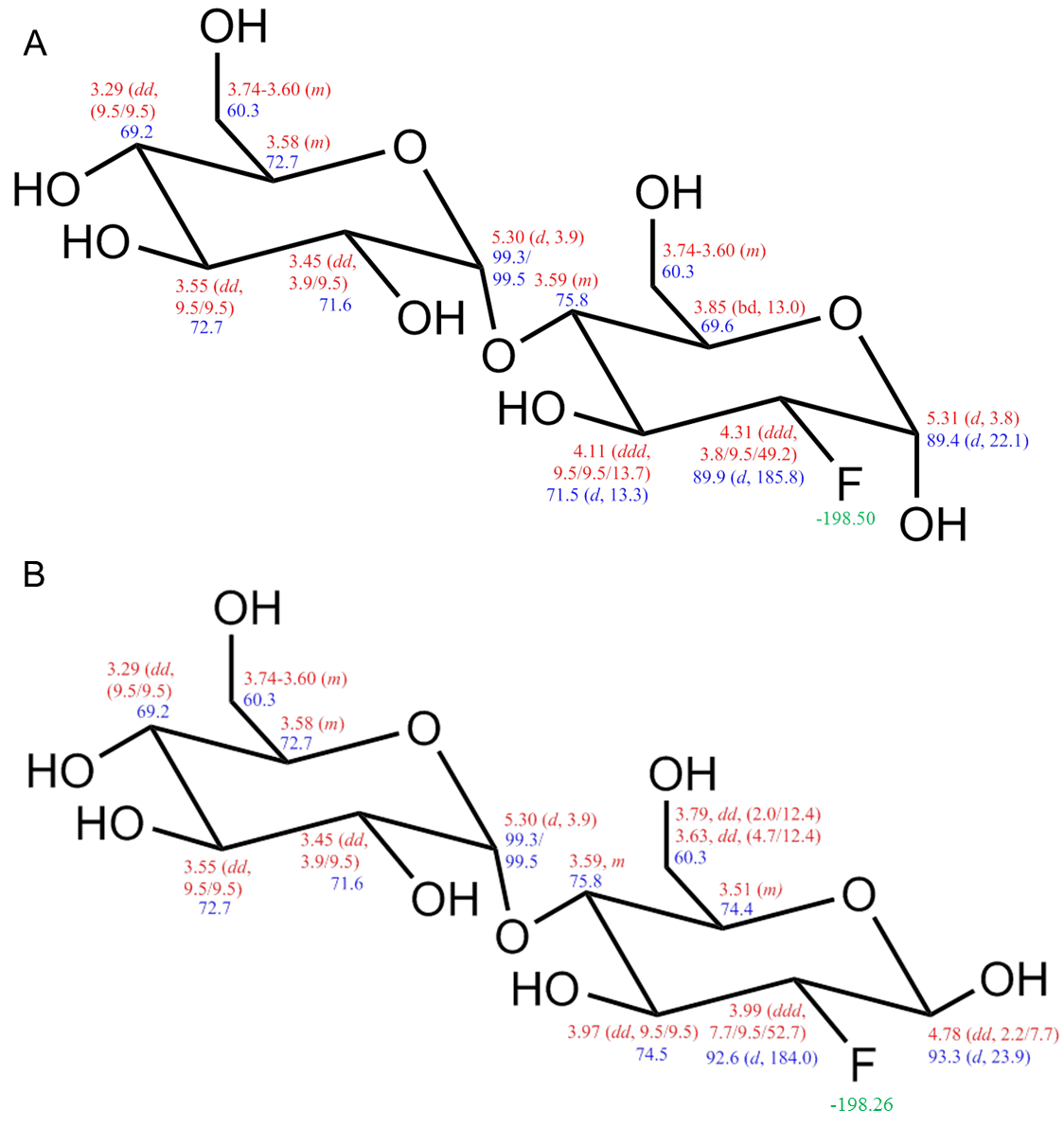

Supplement: Supplementary file 1 [file DataSheet1.DOCX]
